# Debunking Pitfalls of Li–N_2_ Cells for Ammonia Electroproduction: Is This Setup Affordable to Prove Nitro-Fixation before Lithium Plating?

**DOI:** 10.1021/acselectrochem.5c00402

**Published:** 2025-11-15

**Authors:** Anna Mangini, Alberto Garbujo, Pierdomenico Biasi, Valentina Testa, Maria Concetta Bruzzoniti, Luca Rivoira, Sara Garcia-Ballesteros, Federico Bella

**Affiliations:** † Department of Applied Science and Technology, 19032 Politecnico di Torino, Corso Duca degli Abruzzi, 24, Turin 10129, Italy; ‡ Basic Research Department, Casale SA, Lugano, Via G. Pocobelli 6, Lugano 6900, Switzerland; § Department of Chemistry, 9314 Università degli Studi di Torino, Via P. Giuria 5, Turin 10125, Italy

**Keywords:** nitrogen electroreduction, lithium−metal cell, ammonia electrosynthesis, quantification pitfalls, cyclic voltammetry

## Abstract

The Li–N_2_ cell represents a fascinating device that opens a new pathway for ammonia electrosynthesis. It combines the unique property of lithium, which can spontaneously react with N_2_ under mild conditions, with an energy-efficient solution to the challenging N_2_ fixation reaction. However, such a battery-inspired setup may be susceptible to false-positive results and present some pitfalls. This work elucidates some critical aspects of Li–N_2_ cells, aiming at identifying a reliable methodology to assess the electrochemical reduction of N_2_ at the cathodic surface, avoiding misleading pathways. Despite the spontaneous nature of the reaction between lithium and N_2_, it remains uncertain whether it is feasible to promote the electrochemical fixation of N_2_ before reaching the lithium plating potential. This would involve lithium as an ion in the electrolyte, which should activate and enable N_2_ reduction on the carbonaceous surface before any Li^+^ reduction occurs, i.e., at a potential higher than the lithium plating potential (−3.04 V vs SHE). This study discusses this possibility, searching for setup limitations, such as the presence of metallic lithium at the anode, and pitfalls, such as the use of cyclic voltammetry in different testing environments as a methodology to evaluate the formation of Li_3_N before lithium plating occurs.

## Graphical abstract

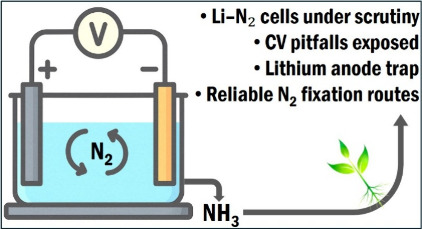

## Introduction

Since the Haber–Bosch process enabled industrial NH_3_ synthesis in 1910, the world population has almost doubled as the amount of nitrogen available for crops, limited by natural rhythms of bacterial fixation, was overcome with synthetic fertilizers.^1–3^ However, the secular Haber–Bosch process requires centralized facilities to enable higher efficiency since the main reaction, i.e., N_2_ thermochemical reaction with molecular hydrogen, requires >200 atm and highly pure gas streams.^4^ The huge amount of H_2_ needed raises concerns about the feasibility of coupling this plant with water electrolyzers, e.g., for the amount of critical raw metals required for these electrolyzers.^5^ Indeed, nowadays, H_2_ is obtained from steam reforming, one of the most challenging processes in terms of decarbonization.^6^ Moreover, a decentralized process could alleviate the socioeconomic inequality in developing countries.^7,8^

Electrochemical routes emerged as an intriguing possibility for the production of NH_3_, especially considering the possibility of working under mild conditions as well as being powered by renewable energy sources. However, the initial enthusiasm in the electrochemical nitrogen reduction reaction (E-NRR) field, which is hampered by the competitive reduction of H^+^,^9^ quickly vanished after the key publication of Andersen et al., who highlighted critical issues in several publications reporting successful E-NRR. Their work revealed that the detected NH_3_ often originated from the conversion of nitrogen oxide impurities present in the N_2_ gas used for experiments, rather than from effective N_2_ reduction. This finding demonstrated that the desired N_2_ fixation was not sensibly achieved.^10^ The possibility of achieving N_2_ fixation through an electrochemical reaction has then been reopened thanks to the application of lithium as a mediator in an aprotic environment.^11^ This alkali metal presents the unique ability to spontaneously react with N_2_ under mild conditions, forming lithium nitride (Li_3_N), which is stable enough to be subsequently protonated into NH_3_.^12^ In just half a decade, significant advancements have been made in the field, achieving 300 h of continuous operation with a Faradaic efficiency as high as 64%.^13^ Current research has delved into the key parameters, such as the solid electrolyte interphase (SEI) layer composition and permeability to the different reactive species, improving the selectivity and stability of the process.^14–18^

On the other hand, the application of a galvanic cell, consisting of a lithium anode and a carbonaceous cathode with an aprotic electrolyte, is aimed at the production of the sole intermediate of NH_3_ electrosynthesis, i.e., Li_3_N; this approach represents an interesting opportunity in the E-NRR field, and the resulting Li_3_N would then be reacted with water to give NH_3_. This strategy bypasses the necessity of using H_2_ as a reagent as well as eliminates the dependence on electrolyzers, reducing the correlated energy consumption and critical-raw-material catalyst needs. Such a stepwise strategy involves (i) lithium metal nitridation into Li_3_N, (ii) Li_3_N protonation, and (iii) lithium-ion reduction back to metallic lithium. Given these premises, it is clear why galvanic cells aimed at exploiting the reaction between lithium and molecular nitrogen, i.e., so-called Li–N_2_ cells, are now considered as truly promising devices with potential applications in both energy storage and NH_3_ electrosynthesis.^19,20^ While sharing energy storage principles with lithium–air batteries, which exploit the reactivity of lithium with molecular oxygen (O_2_),^21^ Li–N_2_ cells also represent an innovative strategy toward NH_3_ electrosynthesis, providing a solution to the challenging N_2_ fixation.^22–24^

In this field, it is mandatory to elucidate some critical aspects of the Li–N_2_ technology, requiring further investigation to avoid being overshadowed by the promising advantages of this approach as well as preventing misleading research pathways in the pursuit of breakthroughs. Even if in the recent literature the interest in Li–N_2_ cells is growing,^25,22,26,27^ only one straight and reliable quantification protocol needed to confirm the effective reaction of the highly stable N_2_ molecule has been developed. This protocol consists of quantifying ^15^NH_3_ obtained from ^15^N_2_ electroreduction by means of nuclear magnetic resonance spectroscopy.^10,28^ Moreover, the gap between battery and electrocatalysis communities still prevents a common view on a relevant issue, the selectivity of the desired reaction.^24^ For energy storage applications, reversibility and energy density are crucial parameters, but in the challenging field of E-NRR, the focus should be on identifying the reactions and the mechanisms able to unlock the N_2_ triple bond cleavage.^29,30,11^

Notwithstanding these considerations, the application of a galvanic cell for the nitridation step could enhance the energy efficiency of the process and enable the use of a nonmetallic cathodic material. This particular stepwise strategy would present several advantages. (i) Mechanistic insights: it would deepen the understanding of the N_2_ activation-reaction mechanism and clarify the feasibility of the Li_3_N formation (or other N-containing intermediates) in the presence of Li^+^ without metallic lithium and near-absence of protons, i.e., similar to electrocatalytic studies of E-NRR rather than metal-mediated systems;^31^ indeed, the galvanic cell should, by definition, work at positive cell potentials, higher than the lithium plating one. (ii) Catalyst and electrolyte optimization: it would enable the application of catalysts and different cations in the electrolyte to enhance NH_3_ production and process efficiency, which would be critical to improve energy efficiency, as a lower cell potential could be achieved through a different chemistry, following an approach similar to that currently under study in metal-mediated systems.^32^ (iii) Material sustainability: it would exploit cheaper, abundant, and more durable materials as cathodic electrodes and supports of the active species; indeed, a carbonaceous support may enable a higher specific surface area and consequently open up to higher current density values, as well as improved stability, allowing for longer operation, both of those characteristics being essential for process scalability.

Therefore, unraveling the cathodic mechanism is necessary to critically face some fundamental questions about the Li–N_2_ technology for NH_3_ electrosynthesis. The possibility of observing the desired reduction reaction from the electrochemical technique of cyclic voltammetry (CV) is also discussed in this work. Additionally, the first question that arises when considering this cell is how to quantify and verify the production rate of the cathodic reaction in a setup where metallic lithium at the anodic side could spontaneously lead to chemical NH_3_ production. In this article, the influence of this spontaneous reaction in Li–N_2_ devices has been quantified, and the possibility of discriminating an effective N_2_ reduction at the cathode is critically discussed. Finally, spontaneously formed Li_3_N on the anode surface could be hydrolyzed from impurities or developed H^+^, leaving a passivating LiOH layer. This layer would enhance the electrical insulation at the surface, undermining the stability of the device.^22^ The present work emphasizes the inherent complexities in interpreting Li–N_2_ electrochemical data and suggests the clarification of results toward a rigorous experimental control.

## Experimental Section

### Electrochemical Setup Assembly and Testing

Tests were conducted in different cell architectures, i.e., coin cell CR2032 (MTI Corp.) and ECC-Air from EL-Cell GmbH. Both architectures were used, sealed, and placed in-flow; for the coin-cell architecture, a specific top with a stainless-steel hole plate was used and tested in a N_2_-filled glovebox. In addition, a batch glass cell with a volume of 25 mL was used for the tests done for architecture comparison. In this case, the electrode holder was made of polyether-ether-ketone, with stainless steel contacts covered by the material used as the electrode, and the separator was not present. The cell assembly was composed of a lithium foil, 200 μm-thick, a glass fiber (GF) separator (Whatman GF, 18 mm diameter and 1.55 mm-thick, supplied by EL-Cell GmbH), and a carbonaceous carbon paper (as specified for each test). The carbon paper was purchased from Toray (060), the carbon cloth from AvCarb (1071), and the 5% Teflon-coated carbon paper covered by a layer of microporous carbon from Sigracet (GDL 24 BC). For the test in which platinum gauze (100 mesh, 99.9%, Sigma-Aldrich) was used as an anode in place of lithium, it was previously annealed by a butane torch in air. The diameter of all the components of the stack was 18 mm when the EL-Cell architecture was used and 15 mm when the cell was assembled in the coin-cell architecture. A Li_0.5_FePO_4_ (LFP) reference electrode was used as a reference electrode, as previously reported.^18,33,34^ To this aim, a commercial LiFePO_4_ powder (Aleees) was chemically partially reduced with K_2_S_2_O_8_ (Sigma-Aldrich) as previously reported,^33^ mixed with C_45_ (Imerys, Timcal) and poly­(vinylidene difluoride) (PVdF, Arkema) as a binder, in a 80:10:10 proportion, in 600 μL of *N*-methyl-2-pyrrolidinone (>99.0%, Sigma-Aldrich) thanks to a tip sonicator for 45 min at 20% amplitude, and deposited onto a copper wire (99.999%, Goodfellow Cambridge Ltd.). The electrolyte solution was prepared in a MBraun argon-filled glovebox (with impurity values of <0.5 ppm H_2_O and <0.1 ppm O_2_). Lithium perchlorate (LiClO_4_) or lithium trifluoromethansulfonate (LiCF_3_SO_3_) (99.99%, Sigma-Aldrich) was added to dimethyl sulfoxide (DMSO) and tetraethylene glycol dimethyl ether (TEGDME) (anhydrous, >99.9%, inhibitor-free, Sigma-Aldrich) to obtain solutions with different molarities. The electrolyte made of ethylene carbonate (EC) and diethyl carbonate (DEC), at a 1:1 ratio, with 1 M lithium hexafluorophosphate (LiPF_6_) was purchased from Solvionic (99.9%). For the acid trap (8 mL), a solution was prepared by diluting HCl 37% (Sigma-Aldrich) up to 0.2 M. For the analysis of the NH_3_ collected in the acid trap, the salicylate method^35^ was used with the following chemicals: sodium hydroxide (NaOH, ≥97.0%), sodium hypochlorite (NaClO, 5 wt % active chlorine), sodium salicylate (C_7_H_5_NaO_3_, 99.5%), sodium nitroferricyanide­(III) dihydrate (Na_2_[Fe­(CN)_5_NO]·2H_2_O, 99.0%), and sodium citrate dihydrate (HOC­(COONa)­(CH_2_COONa)_2_·2H_2_O, ≥99.0%).

For the electrochemical measurements, a VSP-3e Biologic potentiostat was used. For the test under gas flow, after the cell was connected to the instrument, the electrolyte was saturated for 30 min in N_2_, and gas was flowed in the cell at 4 mL min^–1^, either argon or N_2_ (purity 99.9999%), and further purified by passing through a commercial filter (Agilent OT3-4). Meanwhile, the cell potential was measured with the open-circuit potential technique. To determine the electrochemical stability of the different electrolytes tested, linear sweep voltammetry (LSV) at 0.1 mV s^–1^ was performed within a potential range going from the open-circuit voltage (OCV) to 0.5 V vs Li^+^/Li, in ECC-Std test cells (EL-Cell GmbH) composed of the GF separator, impregnated with 500 μL of electrolyte, between a plain stainless-steel piston, used as the cathode, and a lithium metal foil, used as the anode. To observe the stability over time of the electrolyte coupled with the lithium foil, electrochemical impedance spectroscopy (EIS) tests were performed in the same setup between 100 kHz and 0.1 Hz at open-circuit potential and at different times after rest periods. To calculate the electrochemical surface area of the different cathodes, symmetrical cells were assembled in the ECC-Std architecture. The double layer capacitance was obtained by measuring CVs at different scan rates (from 20 to 100 mV s^–1^) in a non-Faradaic potential window of 0.2 V. For the calculation, the medium-specific capacity of carbons in the aprotic electrolyte was used, i.e., 13 μF cm^–2^.^36^ To evaluate the N_2_ reduction reaction, CV tests were carried out on the different cells between 0.5 and 4 V vs Li^+^/Li. An EIS test between 100 kHz and 0.1 Hz was carried out at open-circuit potential before the test and repeated after the CV. The NH_3_ production was investigated by performing galvanostatic constant current (discharge) tests at 0.1 mA cm^–2^. In particular, after the saturation period, LSV was conducted from the OCV to 1.5 V vs Li^+^/Li. Then, the current imposition was alternated with rest periods of 1 min until 1 C of total charge was passed, after which an EIS measurement was performed, the cell disconnected, and the hydrolysis carried out by adding H_2_O inside the cell. For the test conducted with the reference electrode, the internal resistance required for iR correction in the LSV was determined by the EIS technique, and the iR correction was manually compensated. The contribution of the NH_3_ registered in the electrolyte and in the acid trap was summed up to calculate the total production. A blank test was performed with the same procedure, but flowing argon gas instead of N_2_, and the amount obtained was subtracted from the result obtained with N_2_. Scanning electron microscopy images were collected by using a Phenom ProX instrument with a CeB_6_ source. X-rays diffraction (XRD) was carried out using a PANalytical X’Pert (equipped with a Cu Kα radiation source) diffractometer. The diffraction profiles were collected with a 2D solid state detector (PIXcel) from 20° to 80° (2θ).

### Ammonia Quantification

The produced NH_3_, which was collected in the acid trap, was quantified by means of UV–visible spectroscopy, following the colorimetric salicylate method, with a methodology already reported in the literature.^35,37^ The detection limit of this method has been reported to be as low as 0.1 mg L^–1^.^35,37^ The peak of the absorbance at 650 nm was measured by a HITACHI U-500 UV spectrophotometer. Calibration curves were prepared each time a new batch of colorimetric reagents for the salicylate method was prepared. Each calibration point was obtained by dissolving NH_4_Cl in the as-prepared acid trap, diluting to the desired concentration, and subsequently basifying the solution with a fixed amount of 4 M NaOH to reach a pH of approximately 11. The method was validated in terms of linearity (*R* ^2^ = 0.9996, using 6 calibration levels from 0.25 to 2 mg L^–1^), sensitivity (95% confidence interval and a quantification limit of 0.2 mg L^–1^), and precision. The intraday repeatability was 4.2%, and the interday repeatability was 4.5% (based on five replicates). The NH_3_ produced in the electrolyte samples collected after the test was analyzed through ion chromatography with suppressed conductivity. In detail, an IC25 chromatographic system equipped with an AS50 autosampler and an EG40 eluent generator (Thermo Scientific Dionex) was coupled to an IonPac CS16 analytical column (3 mm i.d.; Thermo Scientific, Sunnyvale, CA, USA) to promote the separation of the NH_4_^+^ from the matrix. A 25 μL sample loop was used for injection. The eluent was 30 mM methanesulfonic acid, delivered at a flow rate of 0.36 mL min^–1^, and the column compartment was maintained at 40 °C to ensure optimal analyte separation.

Cation suppression was achieved using a Thermo Scientific Dionex Cation Self-Regenerating Suppressor (SRS 300, 2 mm) operating in AutoSuppression Recycle Mode with a CTC-1 connection kit. The suppressor current was set at 32 mA. The suppressors enabled the system to maintain a background conductivity of less than 2 μS, ensuring high sensitivity for ammonium-ion detection. The retention time of NH_4_^+^ was around 8 min. This protocol allows for the reliable quantification of trace levels of ammonium even in matrices dominated by alkali metals, enabling accurate determination under conditions with Na^+^ or Li^+^ to NH_4_^+^ ratios as high as 100:0.01. To reduce the matrix effect, samples were diluted 1:10 with ultrapure water before injection.

The analytical protocol was validated in terms of linearity, sensitivity, and precision. An *R* ^2^ of 0.998 was obtained using 6 calibration levels ranging from 0.03 to 3 mg L^–1^, with a limit of quantification of 0.03 mg L^–1^. The intraday repeatability was 4.9%, and the interday repeatability was 7.1%, calculated over 10 and 3 replicates, respectively. Data acquisition, chromatographic control, and signal processing were performed using Chromeleon Chromatography Data System software, version 6.80 (Thermo Scientific), allowing for both real-time monitoring and comprehensive postrun analysis.

## Results and Discussion

To address the main question of this work, i.e., whether the configuration of a Li–N_2_ cell is adequate to verify the electrochemical reduction of N_2_ using lithium ions in an aprotic electrolyte without reaching the plating of lithium, two main approaches were evaluated. The first involved electrochemical characterization of the device, specifically the observation of a reductive peak associated with Li_3_N formation. The second focused on the quantification of the end-product, NH_3_, generated in the electrochemical cell. Different limitations and pitfalls were identified in both approaches.

### Electrochemical Study of Li_3_N Formation: Is It a Critical and Reliable Assessment?

The validation of Li_3_N formation in galvanic cells through electrochemical characterization, i.e., observing the reduction reaction at a certain potential higher than 0 V vs Li^+^/Li, has been proposed in the literature. Some studies observed a current density increase in CV, accompanied by an additional peak when the cell was exposed to N_2_ compared with Ar.^19,22,39,40^ A reduction peak at a potential of about 1 V vs Li^+^/Li is common for carbonaceous materials, and it is correlated to Li^+^ reduction and intercalation.^19^ However, the additional peak observed under N_2_ has been claimed as proof of Li_3_N formation.^22^ Nevertheless, it is still unclear whether it is possible to promote electrochemical N_2_ fixation before reaching the potential of lithium plating. Indeed, the reaction between Li and N_2_ is spontaneous, but it has only been verified by using the isotopic labeling protocol in the presence of metallic lithium.

Given these premises, in the present work, different cell components and configurations have been screened to find the best fit for the Li–N_2_ concept applied to NH_3_ electroproduction.

At first, the electrochemical characterizations were performed in a holed-top coin-cell setup. In this architecture, different lithium salts and solvents were tested to identify a stable electrolyte. Then, different carbonaceous materials were compared as the cathodic gas diffusion electrode (GDE), and cell architectures were also tested to maximize the N_2_ availability at the cathodic interface. Finally, the reliability of using CV to assess the activity of galvanic Li–N_2_ cells aimed at forming Li_3_N before lithium plating is discussed. To this aim, different testing environments employing N_2_ or argon gas as a feed were compared.

### Electrolyte Selection

To select the proper electrolyte composition, different solvents and salts commonly used in lithium–air batteries were selected and subjected to chemical and electrochemical stability tests. Regarding solvent screening, DMSO and TEGDME were considered. As lithium salts, LiClO_4_ and LiCF_3_SO_3_ were chosen. Lithium bis­(tri­fluoro­meth­ane­sulfon­yl)­im­ide (LiTFSI), a salt commonly used in Li–O_2_ batteries, was excluded to prevent interference from its degradation products, which could introduce an external source of NH_3_ due to the presence of nitrogen in its structure, potentially leading to inaccurate quantification of process yields and efficiencies. Additionally, the most typical electrolyte for lithium-ion batteries (LIBs), i.e., EC:DEC 1:1, with LiPF_6_ = 1 M, was also tested, as it is commonly known for its electrochemical stability. However, this electrolyte was excluded after the first preliminary test (Figure S1), as it was not suitable for in-flow cells. Indeed, the gas stream led to fast cell drying, and a decreasing current over cycling was observed in CV.

Among the tested salts, LiClO_4_ was discarded, as it favored anodic lithium surface degradation due to its enhanced chemical and electrochemical reactivity. Indeed, a darkening of the lithium surface was observed on a lithium foil covered with a fiberglass separator soaked in 1 M LiClO_4_ in TEGDME and then left in a N_2_-filled glovebox for 5 days (Figure S2). Additionally, the same lithium darkening was noticed after LSV was performed on a cell composed of an inert working electrode, i.e., a stainless-steel piston, with lithium foil as the anode, in the EL-Cell setup. This electrochemical characterization was used to test the electrolyte stability window at reductive potentials (Figure S2). However, this darkening was not observed in similar tests when 1 M LiCF_3_SO_3_ in TEGDME was used as the electrolyte (Figure S2). The registered interfacial reaction was then linked to the inferior stability of the perchlorate anion, which could lead to the evolution of O_2_. The evolution of gas was also observed in a closed coin-cell setup with the same cell composition after CV at 0.1 mV s^–1^.

Regarding the solvents, DMSO showed inferior chemical stability, as self-decomposition of the solvent in the presence of a metallic lithium electrode was observed via EIS measurements at different rest times, conducted in sealed coin cells, assembled in a N_2_-filled glovebox with a lithium anode and an inert stainless-steel cathode (Figure S3). However, the internal resistance of the cell was higher when TEGDME was used. The two solvents were then studied in the semibatch setup, flowing either N_2_ or argon in the gas chamber and through the cathode, which was a GDE made of carbon paper foil. The systems were studied by LSV at 0.1 mV s^–1^, followed by three chronopotentiometry tests. Regarding the latter technique, a constant current of −0.05 mA cm^–2^ was applied for 10 min for each repetition. No substantial differences were observed between the two gases, as shown in Figure S4. However, it is noteworthy to stress that, with DMSO, a higher current was recorded in the cell, and the registered plateau presented a slightly higher potential, i.e., 1.5 V vs Li^+^/Li instead of 1.2 V vs Li^+^/Li. These observations were correlated to the electrolyte decomposition at the carbon paper interface, independent from the nature of the flowed gas, as also suggested from the CVs under argon and N_2_ (Figure S5). Darkening of the electrolyte was also observed for both gases. DMSO was then excluded, as electrolyte degradation, highlighted by the brownish color of the electrolyte, was more evident.

In conclusion, these tests allowed the selection of 1 M LiCF_3_SO_3_ in TEGDME as the electrolyte to be used to assess the electrochemical formation of Li_3_N in this study. The reductive current and the onset potential of different reactions were then investigated through CV. As previously mentioned, an additional reductive peak was expected in CV experiments conducted under a N_2_ environment, in comparison with argon.

### Different Materials, Same Story

Three different cathodic materials were selected to compare three different types of GDEs: one with woven fibers and two composed of nonwoven fibers, with or without a microporous layer. For the woven fiber, a carbon cloth without any microporous layer was chosen. As nonwoven carbon paper (CP), a 5% Teflon-coated material was selected. The third material was a 5% Teflon-coated carbon paper covered by a layer of microporous carbon (GDL). In this last case, the microporous layer should increase the active surface area and store a higher amount of the product, in addition to controlling the wettability and ensuring good contact with the electrolyte. These materials were tested in holed-cap coin cells by performing CV at a low scan rate of 0.1 mV s^–1^ (Figure 1), searching for both the more stable combination and an additional reduction peak attributable to Li_3_N formation.

**Figure 1:**
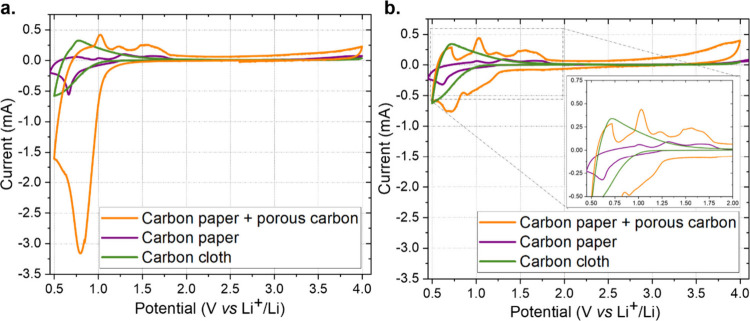
Comparison of different cathodic materials. CVs were measured at 0.1 mV s^–1^ on holed-cap coin cells, tested in a N_2_-filled glovebox, assembled with a lithium anode, 1.55 mm-thick GF separator soaked in 1 M LiCF_3_SO_3_ in TEGDME, and different GDEs as reported in the legend (GDL for the orange curve, the cloth for the green curve, and the CP in the purple curve). (a) First CV cycle and (b) second cycle for each material, with a zoom of the *y*-axis (i.e., the registered current value) in the inset.

As depicted in Figure 1, the currents registered in the reductive scan in the first cycle were higher in comparison to the curves obtained from the second cycle, for both GDL and CP cathodes. In contrast, the carbon cloth, which presents larger woven fibers and shows a lower conductivity and specific surface area, showed the same behavior in the first and second cycles. The reductive peak observed in CP and GDL exclusively during the first cycle was attributed to electrolyte degradation at the cathodic interface and the formation of the SEI layer, as often reported in LIB studies.^41^

To support this hypothesis, which correlates the first sharp peak measured in the CVs with SEI layer formation, different cross-check tests were performed. As shown in Figure 2a, the reactivity of GDL was approximately one order of magnitude higher compared to that of CP. The differences in the first cycle current density obtained with diverse materials were attributed to their different active surface areas. To prove this assumption, the electrochemically active surface area (ECSA) was evaluated by scanning, at different rates, a potential window of 0.2 V in a non-Faradaic region (i.e., near the cell open-circuit voltage) using symmetrical cells. From this measurement (Figure S6a), it was possible to observe that the capacitive current registered with GDL, which was correlated to the effectively active surface of the material exploitable in the cell, was one order of magnitude higher than that of CP. Moreover, the carbon cloth showed a resistive behavior, confirming the availability of a limited ECSA (Figure S6b). Moreover, the specific capacity of the three compared GDEs, obtained by chronoamperometry at 0.9 V vs Li^+^/Li, confirmed higher reactivity with increasing ECSA, suggesting that the observed phenomenon was a surface reaction (Figure S7).

**Figure 2:**
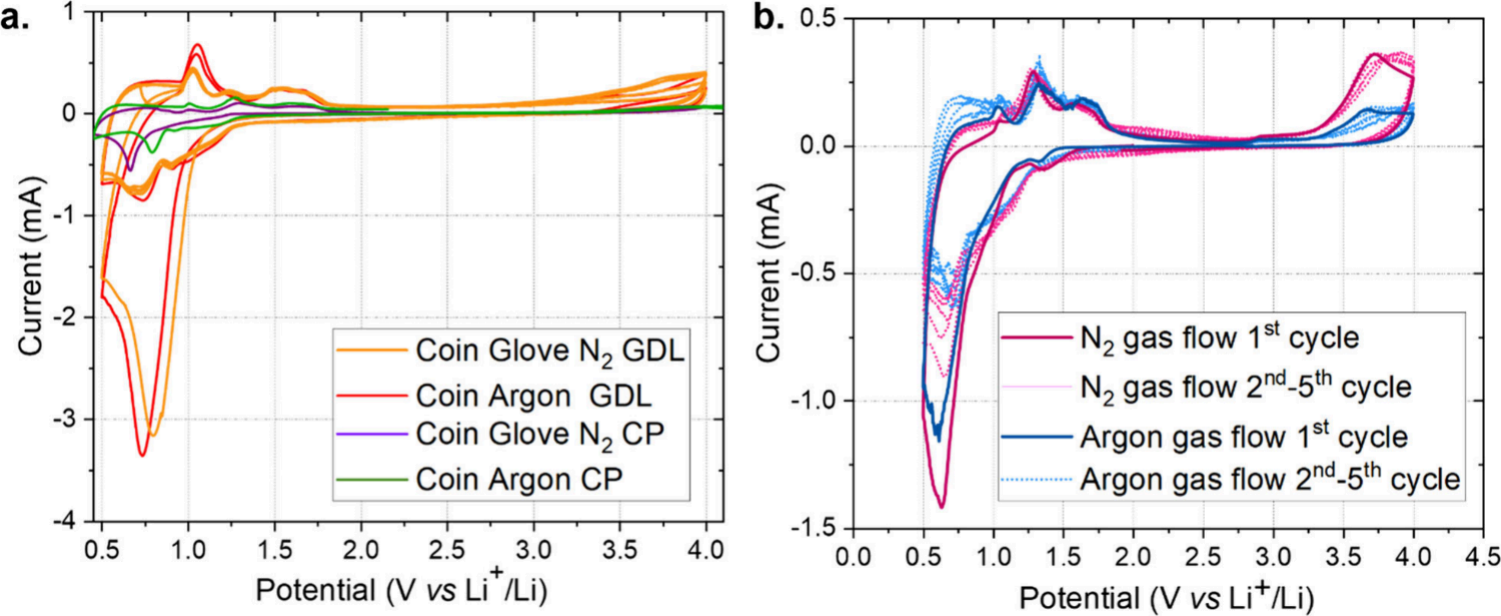
(a) CV traces at 0.1 mV s^–1^ for coin cells with holed caps, tested in different glovebox environments, assembled with a lithium anode, 1.55 mm-thick GF separator soaked in 1 M LiCF_3_SO_3_ in TEGDME, and GDL (red and orange) or CP (pink and green) as the cathode. The cells were tested under argon (red and green) or in a N_2_-filled glovebox (orange and pink). (b) CV traces at 0.1 mV s^–1^ for EL-Cells assembled with a lithium anode, 1.55 mm-thick GF separator soaked in 1 M LiCF_3_SO_3_ in TEGDME, and CP, tested after 30 min of rest (aimed to reach electrolyte saturation), maintaining a constant gas flow of filtered argon (green) or N_2_ (purple) at 4 mL min^–1^.

Taking into account the obtained results, CP and GDL were selected to test the Li_3_N electrochemical formation in the cell. For that purpose, CV experiments under argon and N_2_ atmospheres were completed and compared (Figure 2a). Moreover, the reproducibility of the cells was verified by testing three identical cells. The presence of the sharp reductive peak in the first cycle, also observed in argon, was confirmed as well as the stability upon cycling from the second cycle (Figure S8), even at lower scan rates (Figure S9). The CP showed slight differences between the tests in argon and N_2_ (Figure 2a), and it presented intermediate properties between the three different GDE studies, e.g., a sufficient specific surface area and moderate reactivity with the electrolyte. Therefore, the CP was selected as the cathode for further study of Li_3_N formation in the EL-Cell semiflow architecture, in which the gas flow rate, i.e., 4 mL min^–1^, was better controlled and the N_2_ mass transport should not be a limiting factor. However, the obtained reduction peak onset was the same for both N_2_ and argon flows, and the current density decreased for both gases with cycle number (Figure 2b).

The current peak registered at the first cycle was then related to the formation of a passivating layer on the cathode. The reactions observed from the second cycle with high repeatability were, instead, correlated to interactions of the carbonaceous support with Li^+^, i.e., Li^+^ intercalation and deintercalation. Even if the tests were carried out without a reference electrode, as the lithium anode was assumed as nonpolarizable, the correlation of this measured current with phenomena on the cathode was supported by the absence of this reaction in the LSV of the lithium anode with an inert electrode at the same potential window (Figure S2).

To prove the nature of the first CV cycle reaction peak, EL-Cells were assembled in a N_2_-filled glovebox and then left at their open-circuit voltage for a rest period of 20 days before the potential scan was applied. Then, the cells were tested with the same CV protocol as that used for the previous experiments without any rest period (Figure 3a). These diagnostic tests were designed to verify whether the reductive peak detected in the first CV cycle originated from a spontaneous chemical process or from a purely electrochemical reaction. When the chemical compatibility between the electrode and the electrolyte is insufficient, a spontaneous chemical (non-Faradaic) reaction may occur even before any potential is applied, leading to the formation of a passivation layer at the interface. Such a process can modify the electrochemical response of the system. As a consequence, the reductive process recorded in the CV may appear attenuated, as it could have already occurred partially before the electrochemical measurement. Moreover, EIS can reveal evidence of these interfacial phenomena through the appearance of additional semicircles or an increase in the low-frequency resistance, indicating the formation of new interfacial layers and variations in the internal resistance of the cell.

**Figure 3:**
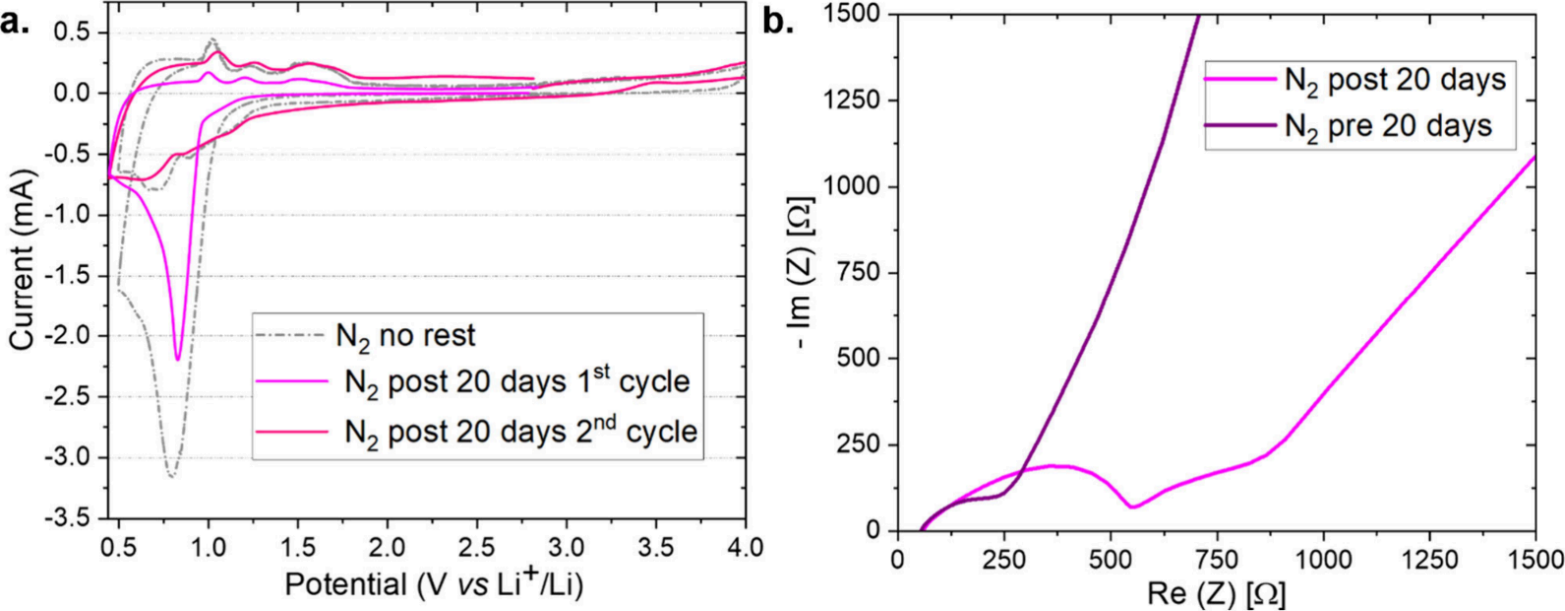
(a) CV traces at 0.1 mV s^–1^ of sealed coin cells assembled with a lithium anode, 1.55 mm-thick GF separator soaked in 1 M LiCF_3_SO_3_ in TEGDME, and GDL. The gray curve is obtained without any rest period, while the pink and red curves represent the first and second cycle, respectively, of a coin assembled under N_2_ and tested with CV after 20 days of rest. (b) EIS measurement of the same cell assembled under N_2_ just after cell assembly (purple line) and after 20 days of rest (pink line); in the latter case, the EIS experiment was performed just before the CV presented in (a).

Accordingly, EIS spectra were recorded both before and after the resting period (Figure 3b). The test was repeated twice in N_2_ and was also repeated in argon, confirming the decrease of the first cycle reductive peak (Figure S10).

The CV traces showed a lower current density at the same onset potential as the previously observed first cycle peak. Moreover, the EIS measurements suggested that an additional layer formed during the resting period, as a new unsymmetrical semicircle was registered after 20 days. These results suggested the occurrence of a spontaneous reaction during the days of rest, as well as the preformation of a spontaneous and chemically induced SEI layer, which could inhibit the electrochemical reduction at the first reductive scan, resulting in a less accentuated reduction peak in the first cycle. The second cycle, instead, showed a similar behavior in both cases, with and without the resting period before the electrochemical characterization, suggesting that these passivation layers (i.e., the one electrochemically formed and the partially chemical one) did not influence the subsequent reactions in the cell.

Moreover, comparing the devices assembled in argon vs N_2_ atmosphere (Figure S9), the different radii of the additional semicircle in the Nyquist plot of EIS of the cells after the rest period indicated the formation of a different spontaneous passivating layer in the presence of the two gases. This difference may be related to the crossover of N_2_ to the metallic lithium anode through the separator. Indeed, it should be stressed that the measurements were conducted, in similarity with Li–N_2_ literature, for the whole cell and then without any reference electrode. The lithium foil may be spontaneously nitridated by N_2_, modifying both the lithium anode surface and the species present in the whole device other than the internal resistances. The reaction of the CP surface was also observed by scanning electron microscopy analysis, in which the partial melting of the Teflon coating from the material surface was hypothesized after CV was performed without the rest phase (Figure S11).

To further confirm the hypothesis that the reductive peak observed in the first CV cycle is related to electrolyte reduction instead of N_2_ conversion, a cell was tested with a plain foil of copper as a cathode. This material was chosen to avoid secondary reactions and should be stable in the selected potential window. The obtained result (Figure S12) showed a notably inferior reductive current, as expected. From the second cycle, the reductive current was really low, suggesting that, at the selected scan rate (0.1 mV s^–1^), the N_2_ reduction on copper was near-negligible or not measured by this analysis. However, even in this case, in the first cycle, a (small) reductive current was registered, and two distinct peaks were observed. The unattended peak in the first cycle may be related to the reduction of the spontaneously oxidized copper surface. Moreover, the reduction on the electrode surface of impurities contained in the electrolyte, e.g., H_2_O, may lead to the peak at 1.5 V vs Li^+^/Li.

### Effect of Impurities on the Electrochemical Characterization

The effect of impurities coming from the cell components and the setup itself was then deepened to assess the relation of the registered reductive current with non-negligible, and often uncontrolled, factors. A test in the semibatch setup was performed using dried air as a feed, instead of N_2_ or argon, further evidencing that the CV technique is not unequivocal for N_2_ conversion studies through this system (Figure 4a). The copresence of O_2_ in the inlet stream was observed from the CV traces, as from the second cycle, and repeated also in further cycles, an additional peak was registered at 2.7 V vs Li^+^/Li, which may be related to the formation of Li_2_O_2_.^42,43^ The limited ECSA of the carbon paper cathode used may limit the available active sites for Li_2_O_2_ formation.

**Figure 4:**
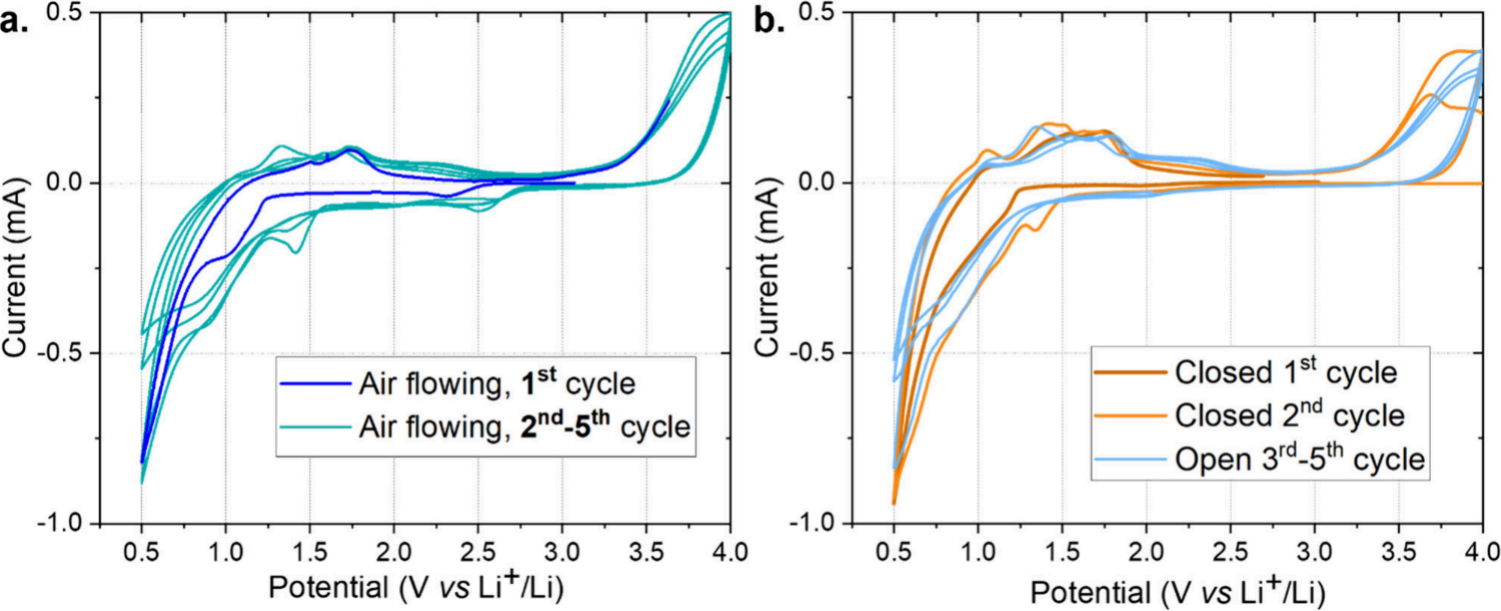
CV traces at 0.1 mV s^–1^ for EL-Cells assembled with a lithium anode, 1.55 mm-thick GF separator soaked in 1 M LiCF_3_SO_3_ in TEGDME, and CP, tested by (a) flowing dry air with a controlled flow rate of 4 mL min^–1^ or (b) closing the cell after the saturation period in N_2_ of 30 min, operating the first two cycles with the cell closed (orange and light orange lines, respectively), and reopening the cell for the third to fifth cycles (light blue lines).

The sources of the impurities were investigated. A low content of impurities from the cell components, e.g., from the electrolyte, could be possible but highly improbable, as all the components are stored in an argon-filled glovebox with monitored impurity values. As the gas stream was filtered and the setup was sealed, there should not be any source of O_2_ or H_2_O from the setup. However, the back-diffusion of air, i.e., from the outlet pipe back into the cell, could not be excluded, as the gas stream was set at a value as low as 4 mL min^–1^. To limit this phenomenon, the internal pressure inside the cell was forced to some millibar thanks to the addition of a liquid column in which the outlet gas was bubbled, creating a back-pressure inside the cell. An acid solution was used as a liquid in this column, aimed at collecting the eventual NH_3_ present in the outlet gas. A test was also conducted using dry air instead of N_2_ (Figure 4a), and it did not reveal distinguishable peaks.

Another unconsidered source of impurities could be the subproduct inside the cell itself. It is known that SEI layer formation could result in both solid and gaseous byproducts, e.g., H_2_ and CO_2_.^21^ These gases, once formed inside the device, could further react in subsequent cycles, creating different passivation layer compositions or leading to further cell degradation. To verify the influence of these gaseous subproducts, CV was performed in the semibatch setup, closing the cell for the first two cycles (Figure 4b). In this case, N_2_ gas was flowed into the cell for 30 min to saturate it. Then, the cell was closed for the first and second CV cycles. After that, for the third cycle, the cell was reopened to the N_2_ gas flow. The obtained traces, reported in Figure 4b, showed an additional reduction peak in the second cycle, while after reopening the cell, the registered trace was similar to the sharp reduction peak observed in previous tests in the semibatch setup.

In the selected setup (the semibatch cell), the presence of the gas stream at the back of the GDE should facilitate the stripping of the gas subproduct from the device, avoiding this domino effect. Different CVs obtained in different cell setups were compared to observe discrepancies between the semibatch architecture and three other setups (Figure S13). The semiflow cell was tested both with and without the liquid column as back-pressure and the semibatch cell with the back-pressure. The obtained CV traces were compared with (i) a coin cell filled with 0.25 mL of electrolyte and with a holed top, through which the N_2_ may enter, but in a static environment, i.e., a N_2_ glovebox with rigorously checked O_2_ and H_2_O amounts, less than 0.5 ppm; (ii) a batch cell filled with 10 mL of electrolyte and tested in a N_2_-filled glovebox; and (iii) a sealed coin cell assembled and closed in N_2_ and filled with 0.25 mL of electrolyte. The N_2_ amount in this last case was further limited, both by the low solubility of N_2_ in the electrolyte (as in the setup described in points (i) and (ii)) and by the inferior void volume containing N_2_.

From the CV curves, only slight differences emerged among the majority of the tested setups, notwithstanding the significant difference in N_2_ availability between static and semiflow setups. In particular, the current density and offset potential registered for the reductive peak were very similar for all setups, except for the flow cell without the back-pressure, for which a shifting of the onset potential of the reduction peak was observed from about 1.5 to 1.25 V vs Li^+^/Li. This result suggested an effective stripping of the produced gas in the case of the semiflow cell without any obstacle for the outgoing gas.

It should be stressed that to ensure good contact of the cathode with both the gas and the electrolyte, flooding of the electrode should be avoided. In the tested setup, flooding was supposed to be avoided, as the amount of electrolyte in the separator (500 μL for a 1.55 mm-thick GF separator) was selected to wet the separator without exceeding its volume. However, as the cell was closed and due to the fact that the cell stuck was not visible inside the cell case, it could not be directly controlled if any electrowetting of the carbon paper enhanced the flooding of the cathode, considering also that the material was teflonated and the hydrophilicity of the surface may not have ensured the electrolyte retention outside from the gas chamber. Nevertheless, the gas stream was supposed to favor drying of the separator, preventing flooding of the electrode.

To summarize, the electrolyte composition and cathodic material were selected after an initial screening. Then, different CVs were measured under N_2_ or argon, but no substantial differences were observed in support of the electrochemical Li_3_N formation. Moreover, the observed reduction peak present only in the first cycle was supposed to be related to the electrolyte decomposition in the first cycle and the subsequent reduction of gaseous subproduct in the cell. The reductive peak may also be related to reactions of impurities, which are characterized by an onset potential similar to that of the desired reaction of Li_3_N formation. Therefore, the CV appeared not sufficiently univocal toward the identification of electrochemical N_2_ fixation in galvanic cells.

### Lithium Anode Interference

N_2_ crossover to the lithium anode has already been discussed in the literature,^22^ and the quantification of NH_3_ in the anolyte in a two-compartment cell was verified.^27^ Moreover, the diffusion of produced NH_3_ in the electrolyte has been assessed.^44^ However, the possibilities and drawbacks of using metallic lithium as an anodic material, similar to lithium–air batteries, have not been critically discussed yet. The use of metallic lithium, as previously explained, presents many advantages in a complete process. However, at the present research stage, the presence of metallic lithium could hinder the interpretation of correct results, as the isotopic labeling cannot distinguish between cathodically or anodically produced NH_3_. Indeed, in this case, even the use of ^15^N_2_ would be converted into ^15^NH_3_ both at the anode and at the cathode, again resulting in the impossibility of separately quantifying the effects of metallic lithium and the reduction reaction.

In this study, the passivation of lithium was observed in the EL-Cell semibatch architecture and analyzed with both XRD and through the quantification of the produced NH_3_. After the CV tests in N_2_, which were about 4 days long, the darkening of the surface of the lithium foil at the anode highlighted the N_2_ crossover through the cell (Figure S14). Even if the N_2_ mass transport was limited by the solubility in the electrolyte and slowed by the presence of the separator, the N_2_ crossover was also supported by the air-free XRD analysis of the postmortem lithium anode foil (Figure S14). The obtained pattern corresponded to the LiOH standard, which could be a residue of protonated Li_3_N, together with the pattern typical of the spectra of Li and Li_2_O.

To verify the hypothesis of N_2_ crossover to the lithium anode, both direct nitridation tests of lithium with N_2_ and production tests were conducted in the semiflow cell setup. The former aimed at assessing the time necessary for this spontaneous reaction to happen in the selected setup, and the second aimed at quantifying the difference in NH_3_ production with and without a lithium anode.

### Direct Lithium Nitridation

For the direct lithium nitridation tests, a lithium foil was placed alone in the same semiflow cell setup, and N_2_ was flowed in the cell for different times. The results (Figure 5) showed that 1 h of N_2_ flow at 4 mL min^–1^, followed by 2 h of rest under a N_2_ atmosphere, was insufficient for complete nitridation of the lithium foil. Indeed, despite the observation of characteristic peaks of Li_3_N in the XRD pattern, the peaks of lithium remained predominant. Regarding the sample obtained after 72 h of N_2_ flow, the characteristic peaks of the Li_3_N crystalline structure were clearly visible, while lithium peaks almost disappeared. Moreover, in both samples, the pattern typical of LiOH was slightly visible, suggesting partial Li_3_N hydrolysis or metallic lithium secondary reactions with impurities, which could be diffused inside the test setup or encountered during the air-free sample holder preparation or analysis.

**Figure 5:**
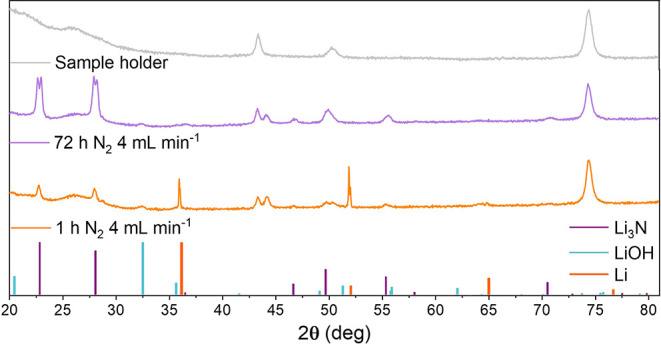
XRD patterns of samples of nitridated lithium in the stainless-steel case for different testing times. From top to bottom: the pattern of the Kapton and stainless-steel holder (gray line), the lithium sample on which N_2_ gas was fluxed at 4 mL min^–1^ for 72 h (purple line), and the lithium sample on which the N_2_ gas was fluxed at 4 mL min^–1^ for 1 h (orange line). At the bottom, the characteristic peaks for different crystalline materials are shown: Li (orange peaks), Li_3_N (purple peaks), and LiOH (light blue peaks). All patterns were plotted after subtracting this background, leading to inconsistencies in intensity between very low and very high angle measurements. Despite these limitations, the analysis was sufficient for the identification of the species present in the sample.

Moreover, the XRD analysis revealed nearly complete nitridation after only 24 h at ambient temperature in the case of a freshly cleaned lithium foil surface. This foil was indeed scratched just before the test to remove a possible passivation layer present on its surface, i.e., a spontaneously formed layer, unavoidably even in the inert storing environment of the argon-filled glovebox.

The reaction of lithium in a complete electrochemical device, however, could be substantially different due to two main reasons: (i) the surface in that case could be passivated by a spontaneous reaction of lithium with the electrolyte; (ii) the presence of the electrolyte and the other cell components could drastically slow the N_2_ diffusion to lithium. Moreover, the formation of nitridated species in the copresence of the electrolyte could lead to the spontaneous reaction of these species with protons from the electrolyte, converting these surface species into NH_3_ or other products dissolved in the electrolyte. These hypotheses were supported by the less severe darkening of the lithium foil observed when used as an anode in CV, which was registered both by visual inspection and by XRD analysis (Figure S14).

### Lithium Direct Nitridation during E-NRR Test

The spontaneous reaction between the anodic lithium foil and N_2_ diffused to this electrode could completely hinder the effective E-NRR reaction at the cathode since the amount of chemically obtained NH_3_ at the anode was expected to be higher than that of challenging reaction at the cathode in the studied setup. To test the possibility of subtracting the contribution of the lithium anode in the NH_3_ production with this cell setup, the NH_3_ obtained from a full Li–N_2_ cell was compared to a cell using a platinum mesh as the anode. The same protocol was repeated for both Li–N_2_ and Pt–N_2_ cells.

To test the E-NRR in the selected semiflow cell with 1 M LiCF_3_SO_3_ in TEGDME, a constant current as high as −0.1 mA cm^–2^ was imposed on the device, following a manually compensated LSV to set the cell at the cell potential of interest, 1.5 V vs Li^+^/Li. Constant-current periods of 1 min were alternated with rest periods to avoid excessive polarization of the electrodes. This electrochemical protocol was designed accordingly to the presence of platinum as an inert electrode, as in this case the oxidation reaction of the previously used lithium (i.e., into Li^+^) was substituted with electrolyte oxidation. Moreover, the change of the anodic material made it necessary to verify that the working electrode potential did not overcome the lithium plating potential since the potential of the anode, when platinum was used, was no longer approximable as stable and constant. To measure the potential of the cathode during the test, an LFP reference electrode was added in the semibatch EL-Cell. To this aim, a LFP-coated stainless-steel pin was inserted in the middle of the GF separator, similar to LFP references used in the lithium-mediated NH_3_ electrosynthesis literature.^33,34^ The tests were performed until 1 C of charge passed in the cell. The device was tested by adding an acidic trap after the cell to collect the eventually produced NH_3_ in the outgoing N_2_ flow. Indeed, even if any proton donor was added to the system, the presence of H^+^ in the cell cannot be excluded. For example, protons could be added from adventitious impurities or formed as a subproduct of the electrolyte degradation or SEI layer formation. These protons may react with spontaneously formed Li_3_N at the anode (when lithium was used as the anode) to give NH_3_. As soon as the electrochemical protocol was performed, a hydrolysis step was conducted directly inside the cell to convert the intermediate products into NH_3_, which should remain dissolved in the electrolyte and partially strip the cell from the gas flow (bubbled in the acid trap). The different contributions of the produced NH_3_ for both lithium and platinum anodes are collected in Table 1. The quantification was assessed by an ion chromatography method on the electrolyte samples (examples of the obtained chromatograms are reported in Figure S15) and with the salicylate method and UV–visible analysis for the acid traps. A control test in argon was also performed.

**Table 1:** Produced NH_3_ (in μg cm^–2^ Geometric Electrode Area) Calculated from the Quantification of Samples of Acid Traps and Electrolytes Obtained after Tests Operated in EL-Cell with a Flow Rate of 4 mL min^–1^ ^a^

	Quantified NH_3_ (μg cm^–2^)			
Cell name	Acid trap	Electrolyte	Anode	Cathode
Li Ar	0.3	0.2	Li	CP
Li N_2_	1.7 ± 0.5	3 ± 1	Li	CP
Pt N_2_	0.3 ± 0.2	0.5 ± 0.3	Pt	CP

a The components of the cell and gas used are specified for each test. Three independent replicates were performed for each test condition under a N_2_ flow.

NH_3_ production was one order of magnitude higher when lithium was employed as an anode, verifying the suspected inapplicability of this material for experimental E-NRR assessment. The “positive” influence of the lithium anode was even more accentuated in the CV tests, as those experiments were longer (i.e., about 4 days instead of less than 1 day) (Table S1).

It is possible to notice that the error is not negligible. The electrolyte itself, before the test, as prepared and stored in the argon glovebox, contained about 0.1 ± 0.1 μg cm^–2^ NH_3_, and the tests in argon showed that the acid trap is also susceptible to accumulating NH_3_, probably trapping NH_3_ from the air.

The scarce repeatability when the lithium anode was used could be correlated also to the boosting effect of the current imposition on the direct lithium nitridation: during the oxidation of Li into Li^+^, the surface of the anodic foil could increase its specific surface area and form a more porous and irregular interface exposed to the electrolyte, and than to the N_2_ solved in it. Moreover, the presence of Li_3_N was observed in the LIB study not to drastically passivate the lithium surface, as Li has been supposed to be able to migrate in the Li_3_N.^45^ The scarce reproducibility of the active surface area of the lithium anode made the setup prone to variability from one test to another; therefore, the NH_3_ obtained from the anode is not repeatable enough to exclude a constant contribution.

For these reasons, using lithium as the anode is highly discouraged when studying Li–N_2_ cells aimed at verifying and quantifying the E-NRR at the cathode. To suppress side reactions and adequately study this system, we propose replacing lithium with an anodic material that is inert toward N_2_ activation, such as platinum. This alternative should ideally also act as a cation reservoir, replenishing the electrolyte with Li^+^ ions consumed at the cathode during the electrochemical formation of the azo intermediate. For future studies, an intercalation or conversion host, taking inspiration from materials used in LIBs, could be explored to supply Li^+^ through a well-defined redox reaction, thus avoiding the direct chemical interaction between N_2_ and metallic lithium. Moreover, such substitution could make the process more cost-effective: a different alkali metal that is more abundant, inert toward N_2_, and capable of providing cations with fast kinetics (and hence higher current densities) could be considered. For example, potassium has been reported to exhibit a weaker binding energy with N_2_ and does not spontaneously bind N* under standard conditions.^46^

## Conclusions

Two main critical aspects are highlighted in this work: the unaffordability of electrochemical characterization by CV to demonstrate N_2_ reaction on a carbonaceous cathode and that the quantification obtained with a lithium metal anode resulted in misleading results.

The use of CV as an electrochemical characterization for the verification of Li_3_N formation is critically discussed. Even if this technique has been often reported to support the development of this reaction in the cell, in this study the ambiguous nature of this measurement is demonstrated. Thanks to CV measurements under different setup conditions and cell components, the influence of different uncontrolled factors on the analysis is highlighted, supporting the idea that the CV technique is not sufficiently unequivocal to prove the Li_3_N electrochemical reaction. The technique is shown to be susceptible to false positives due to the presence of impurities and other unaccounted and not easily controlled phenomena such as the formation of subproducts.

The challenging verification of N_2_ fixation through an electrochemical reaction in the Li–N_2_ galvanic cell, i.e., the N_2_ activation on a carbonaceous cathode coadjuvated by Li^+^, turns out to still be an open question.

As a second aspect, the spontaneous reaction between the anodic lithium foil and N_2_ is proved to give a greater NH_3_ yield in comparison with the three-phase reaction at the carbonaceous cathode. Indeed, the NH_3_ produced from a cell with a lithium anode was one order of magnitude higher than that produced from the one with a platinum mesh anode, in which the only source of nitrogen should be the N_2_ flowing gas. Therefore, anodic spontaneous NH_3_ production may hinder the cathodic reaction and its verification. Moreover, the imposition of the current in the cell could enhance the specific surface area of the lithium anode due to the formation of an irregular morphology on the surface during the lithium oxidation into Li^+^, increasing the reactive area available for nitridation and consequently the developed NH_3_. The variability of this aspect makes it very hard to predict and subtract the contribution of the anodic reaction from the total cell.

In conclusion, the results summarized in Figure 6 indicate that, in the studied setup, it was not possible to distinguish the electrochemical N_2_ reduction from electrolyte degradation based solely on electrochemical characterization. Furthermore, the spontaneous reaction of metallic lithium with N_2_, which generates an additional source of NH_3_, prevents accurate quantification of the electrochemically produced ammonia. Therefore, these results suggest taking a step back in this research field, moving the focus from cell optimization to the identification of a proper test and quantification methodology to verify N_2_ fixation at potentials higher than the lithium plating potential.

**Figure 6:**
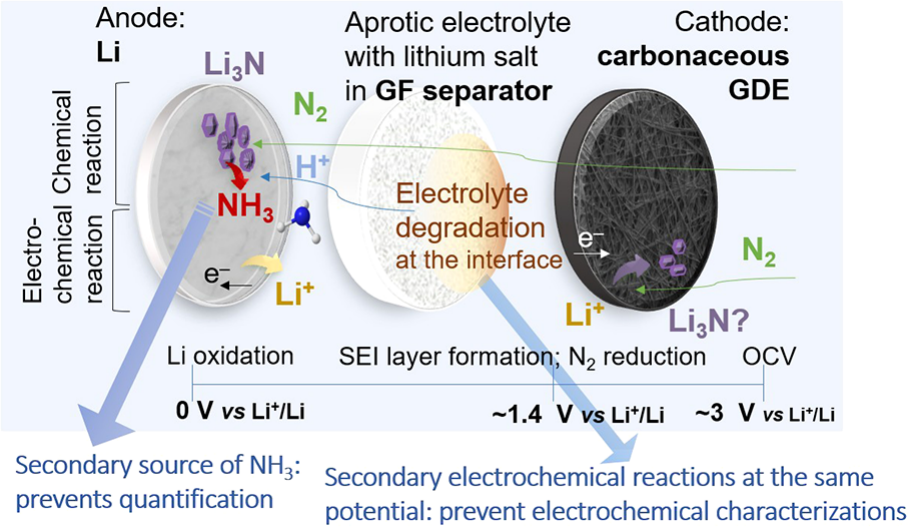
Schematic representation of the chemical and electrochemical reactions of a Li–N_2_ cell (with relative potentials vs Li^+^/Li), pointing out the main limitations of this setup.

A suggested research pathway is the application of a different anodic material, i.e., one different from lithium and inert with N_2_, for the preliminary studies aimed at delving into the verification of NH_3_ electroproduction, e.g., intercalation or conversion materials used in LIBs or a different metal such as potassium.

*Operando* techniques, such as spectroscopy analysis (e.g., surface-enhanced infrared absorption spectroscopy),^47^ differential or online electrochemical mass spectrometry (EC-MS),^48,49^ and synchrotron measurements (e.g., grazing incidence wide-angle X-rays scattering),^17^ can be employed to identify intermediates and products of N_2_ reduction, providing direct *in situ* evidence of their formation. However, the typically low Li_3_N yield, the simultaneous presence of interfering species (e.g., H_2_O in EC-MS analyses of NH_3_),^48^ and the high operational cost limit the routine application of these techniques.

Therefore, a series of faster and less expensive electrochemical diagnostic tests are suggested as preliminary experiments to exclude false-positive results before employing more complex *operando* methods. Conventional electrochemical techniques, such as CV, present pitfalls in the disentanglement of N_2_ electroreduction from side reactions occurring at a similar potential. For this reason, the complementary diagnostic strategies reported in this work are aimed at verifying the electrolyte stability and identifying parasitic processes that may prevail over N_2_ reduction. In addition to conventional tests carried out under argon, the use of purified gases and the assessment of the electrolyte stability window with inert electrodes are recommended. Furthermore, the chemical and electrochemical compatibilities between the electrolyte and the cathodic material should be verified. The spontaneous passivation of the electrode/electrolyte interface was assessed by conducting CV after a resting period of days, studying the interface modifications through EIS, and comparing the results with those of more inert cathodes. To further investigate the formation and influence of side products, experiments involving alternating gas flow and sealed-cell conditions can be conducted.

Finally, to achieve reliable electrochemical Li_3_N formation in those cells, improvements in the cell design are required. For example, it would be precious to increase the electrolyte volume to mitigate the effects of partial electrolyte degradation, and the shielding of the metallic current collector at the cathode (which ensures the electrical connection to the GDE) from the electrolyte to prevent any eventual lithium electrodeposition on its metallic surface. The use of polyether-ether-ketone (PEEK)-based cells is suggested to this aim. Once the cathodic reaction has been reliably verified, further developments could enable the operation of a complete Li–N_2_ galvanic cell. They include the implementation of separators that prevent N_2_ crossover and protect the lithium anode from spontaneous nitridation, combined with a larger-scale setup. The accumulation of a sufficient amount of Li_3_N, subsequently hydrolyzed to NH_3_, is essential to clearly overcome adventitious impurities and demonstrate the practical applicability of this electrochemical process.

## Supplementary Material



## Notes

Additional electrochemical measurements, morphological and structural studies of electrodes, and analytical protocols used for ammonia quantification (PDF)
