# Supplementary material for: Debunking Pitfalls of Li–N2 Cells for Ammonia Electroproduction: Is This Setup Affordable to Prove Nitro-Fixation before Lithium Plating?
Source: ACS Electrochem. 2025 Nov 15;1(12):2866–77. doi: 10.1021/acselectrochem.5c00402 (PMC12683635; doi:10.1021/acselectrochem.5c00402)
Supplement: Supplementary file 1 [file ec5c00402_si_001.pdf]

Supporting Information

Debunking pitfalls of Li-N_2 cells for ammonia electroproduction: is this setup affordable to prove the nitro-fixation before lithium plating?

Anna Mangini,[a] *Alberto Garbujo,*[b] *Pierdomenico Biasi,*[b] *Valentina Testa,*[c] *Maria Concetta Bruzzoniti*[c], *Luca Rivoira,*[c] *Sara Garcia-Ballesteros,*[a,*] *Federico Bella*[a,*]

[a] Department of Applied Science and Technology, Politecnico di Torino, Corso Duca degli Abruzzi, 24, Turin 10129, Italy

[b] Basic Research Department, Casale SA, Lugano, Via G. Pocobelli 6, Lugano 6900, Switzerland

[c] Department of Chemistry, Università degli Studi di Torino, Via P. Giuria 5, Turin 10125, Italy

* Corresponding Authors: sara.garcia@polito.it / federico.bella@polito.it

Contents of SI:

Figure S1: CV traces of Li-N_2 holed-cap coin cells.
Figure S2: LSV measurements of different electrolytes, with photos of a lithium foil.
Figure S3: EIS measurements of coin cells.
Figure S4: LSV and chronopotentiometry at a constant current of cells.
Figure S5: Electrochemical comparison of different solvents.
Figure S6: CV traces for ECSA calculation.
Figure S7: CA for coin cells.
Figure S8: Repeatability of CVs.
Figure S9: Repeatability of CVs with CP and at different scan rates.
Figure S10: Repeatability of CV and EIS measurement.
Figure S11: SEM analysis of post-mortem cathodes.
Figure S12: CV traces of EL-Cells.
Figure S13: Second cycle of CV traces for different cell architectures.
Figure S14: XRD spectra of involved cell components.
Table S1: Quantified NH_3.
Figure S15: Chromatograms of two electrolyte samples.
Bibliography

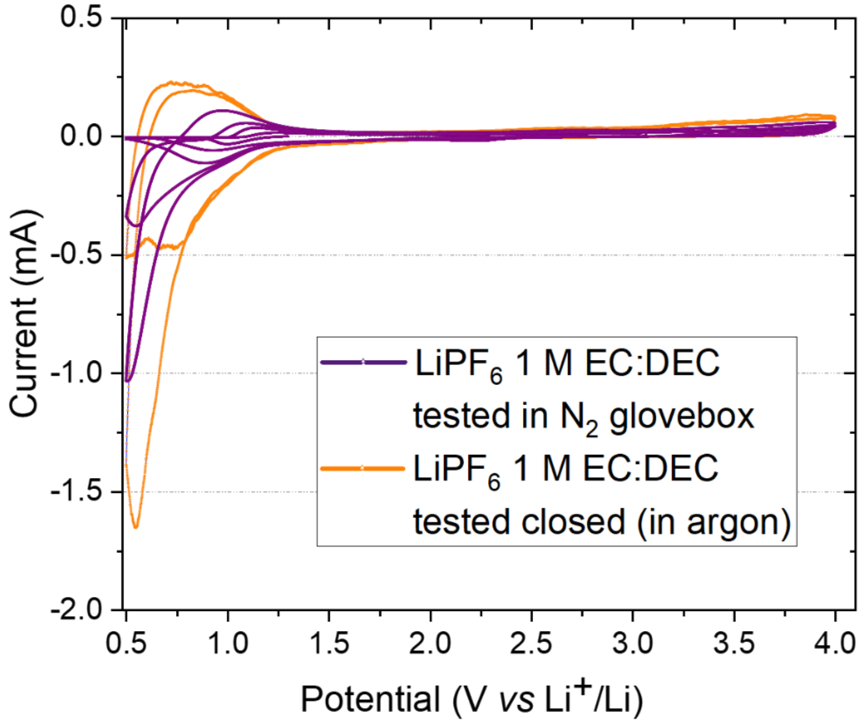

Figure S1: CV traces at 0.1 mV s^{-1} of Li-N_2 holed-cap coin cells, assembled and tested in the N_2-filled glovebox (purple line), or with a closed cap, assembled and sealed in argon (orange line). The cell was composed of a lithium foil as anode, a 1.5 mm-thick GF separator soaked in $LiPF_6$ 1 M EC:DEC, and a GDL cathode.

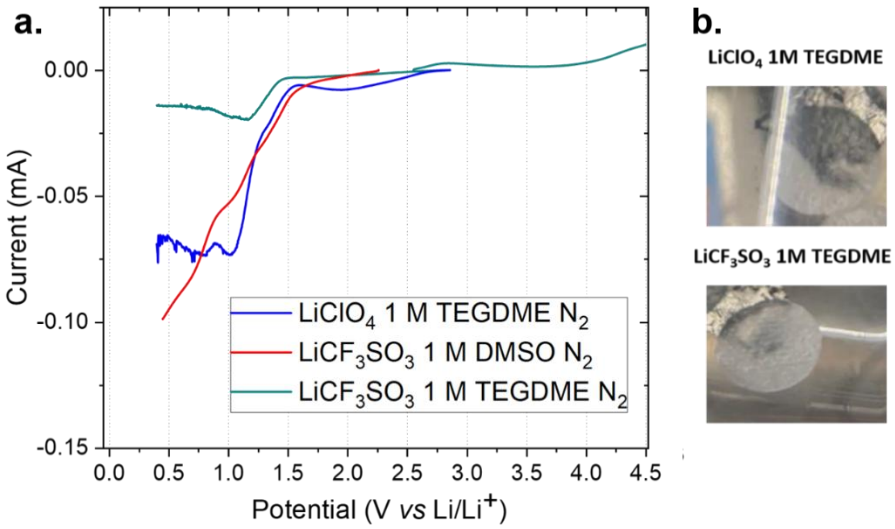

Figure S2: a. LSV measurements at 0.5 mV s^{-1} of different electrolytes, tested in a static EL-Cell setup, with lithium as anode, GF cloth as separator, soaked with different electrolytes, as shown in the legend, and a SS working electrode. The electrode area was 2.54 cm^2. **b.** Photos of a lithium foil stored in the N_2 glovebox after 5 days, covered by a 0.65 mm-thick GF separator, soaked in $LiClO_4$ 1 M in TEGDME (upper picture) or in $LiCF_3SO_3$ 1 M in TEGDME (lower picture).

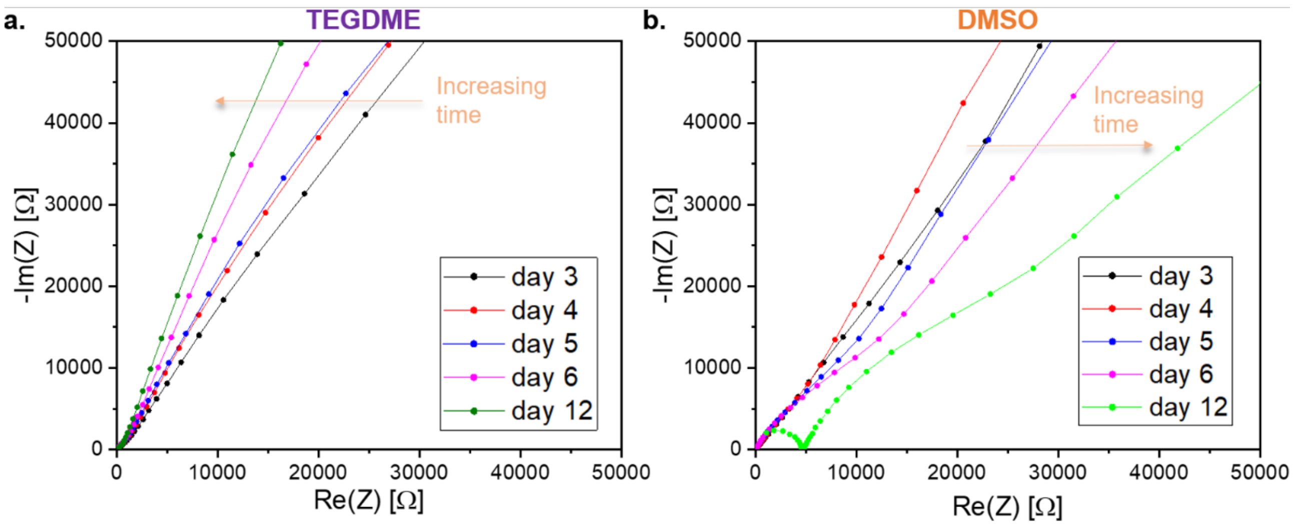

Figure S3: EIS measurements of coin cells composed with a lithium foil as anode, an inert stainless-steel cathode, and a 0.65 mm-thick GF separator soaked with $LiCF_3SO_3$ 1 M in TEGDME (**a**.), or in DMSO (**b**.). The analysis was conducted between 100 kHz and 1 mHz, with a potential amplitude of 10 mV.

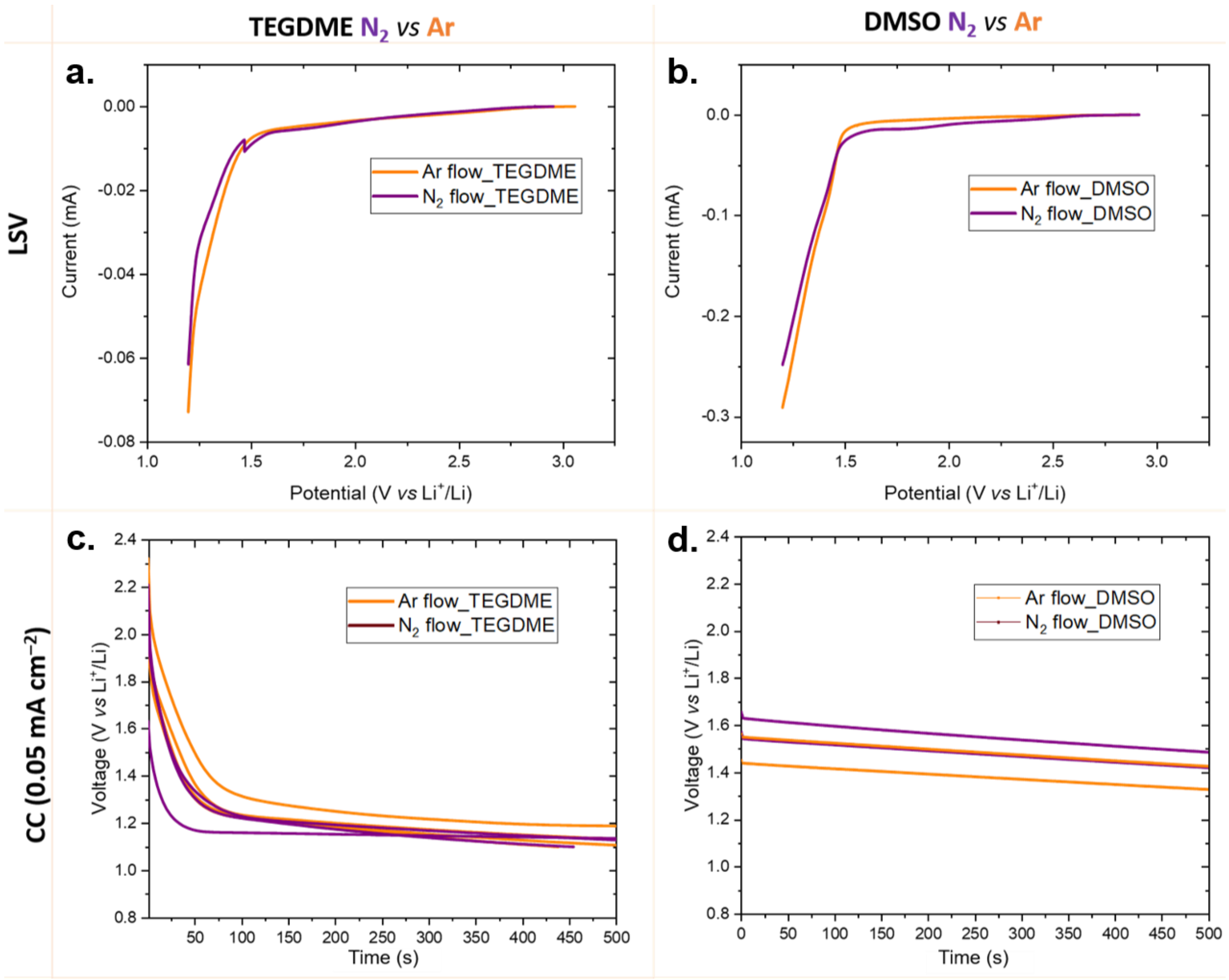

Figure S4: LSV at 0.1 mV s^{-1} (**a.**, **b.**) and chronopotentiometry at a constant current (CC) of -0.05 mA cm^{-2} (**c.**, **d.**) of cells assembled in the EL-Cell semi-flow configuration, with lithium as anode, GF as separator, and CP as cathode. The electrolyte was $LiCF_3SO_3$ 1 M in TEGDME (**a.**, **c.**) or DMSO (**b.**, **d.**). The orange curves correspond to the cell tested flowing argon (4 mL min^{-1}), while the purple ones correspond to N_2 in-flow cells (again 4 mL min^{-1}).

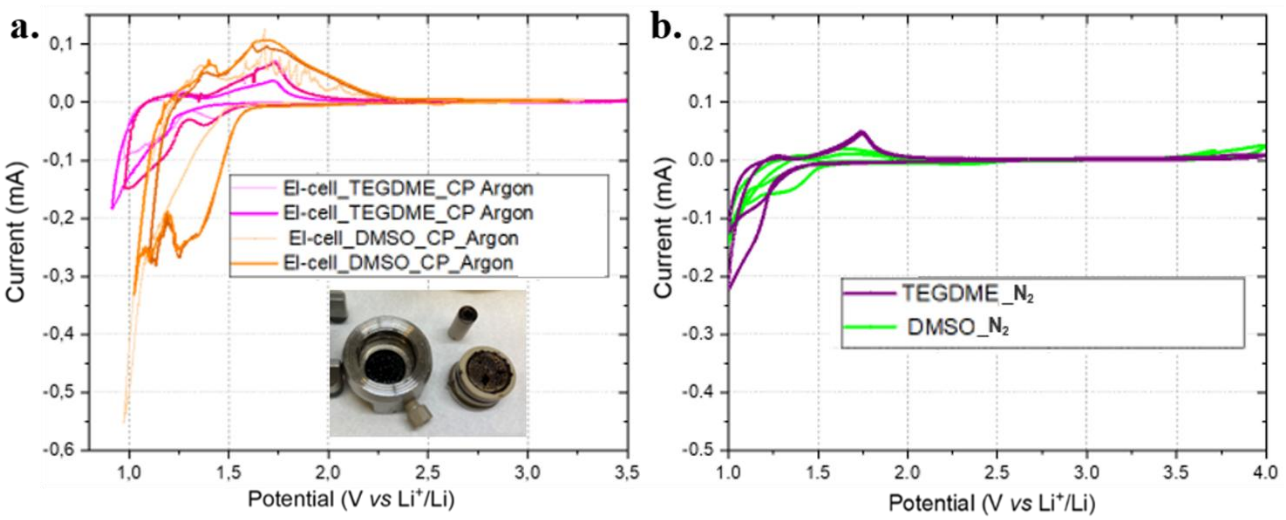

Figure S5: Comparison of different solvents. The CVs were performed at 0.1 mV s^{-1} in the EL-Cell setup, keeping the cell closed after the assembly in argon for **a.**, and, in **b**., flowing the gas, *i.e.*, N_2, with a flow rate of 4 ml min^{-1}, in the cell. The cells were assembled with a lithium anode, a 1.55 mm-thick GF separator soaked in $LiCF_3SO_3$ 1 M in TEGDME (pink and purple line) or DMSO (yellow or green line), as detailed in the legends, and the carbon paper as cathode. In the inset, the darkening of the electrolyte after the CV in DMSO is visible in the re-opened cell.

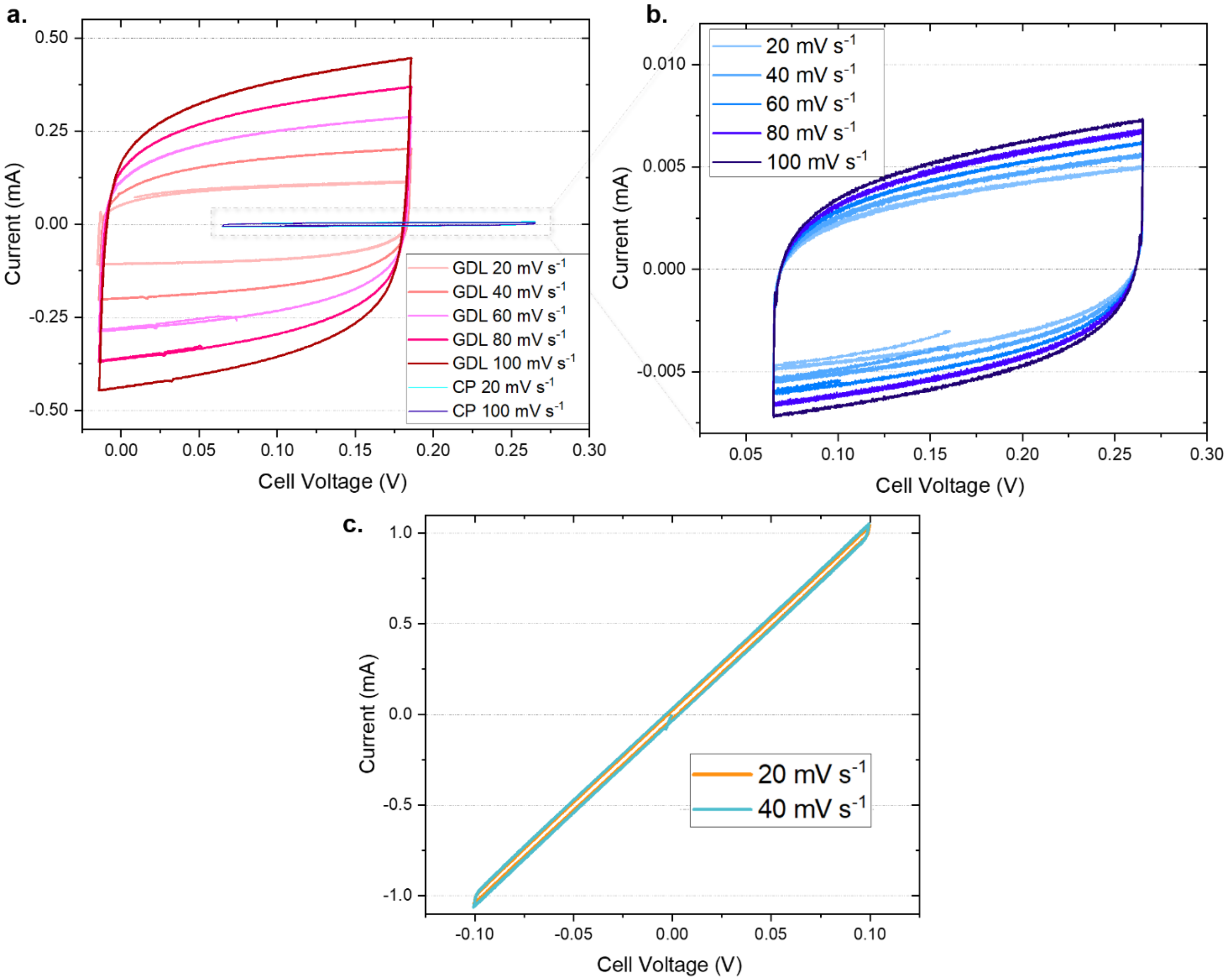

Figure S6: CV traces for ECSA calculation, measured scanning a not-Faradaic area (± 0.1 V *vs* initial potential) at different scan rates. The cells were assembled in the EL-Cell configuration, without any gas flowing (as it was tested not to affect the ECSA), and were composed of two symmetrical electrodes, *i.e.*, different carbonaceous supports, and the 1.55 mm-thick GF separator soaked in $LiCF_3SO_3$ 1 M in TEGDME. The slope of the linear dependency between the delta of the current measured at the middle potential of the scan, and the scan rate itself, was used to calculate the ECSA. **a**. Comparison of the results of ECSA obtained with CP and GDL. CV traces of symmetric cells with two GDL (pink lines) or CP (blue lines) electrodes. The initial potential was fixed at 0.160 V for CP, as it was measured as the OCV of the cells, and at 0.080 V for GDL. **b**. CV traces at different scan rates for CP, corresponding to a zoom of the blue lines in **a. c**. CV traces for ECSA calculation for the carbon cloth, showing a resistive and linear behavior. The initial potential was the OCP of 0.05 V.

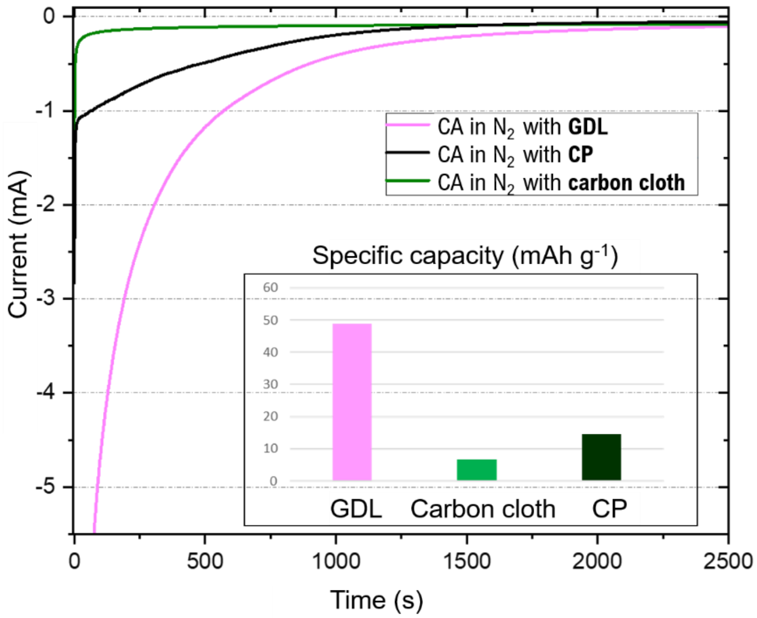

Figure S7: CA at 0.9 V *vs* Li^+/Li for coin cells assembled with a lithium anode, 1.55 mm-thick GF separator soaked in $LiCF_3SO_3$ 1 M in TEGDME, and GDL (pink), CP (black), or carbon cloth (green) as cathode. The cells were tested in a N_2-filled glovebox. In the inset, the resultant specific capacity was calculated and reported for the different tested cathodic materials.

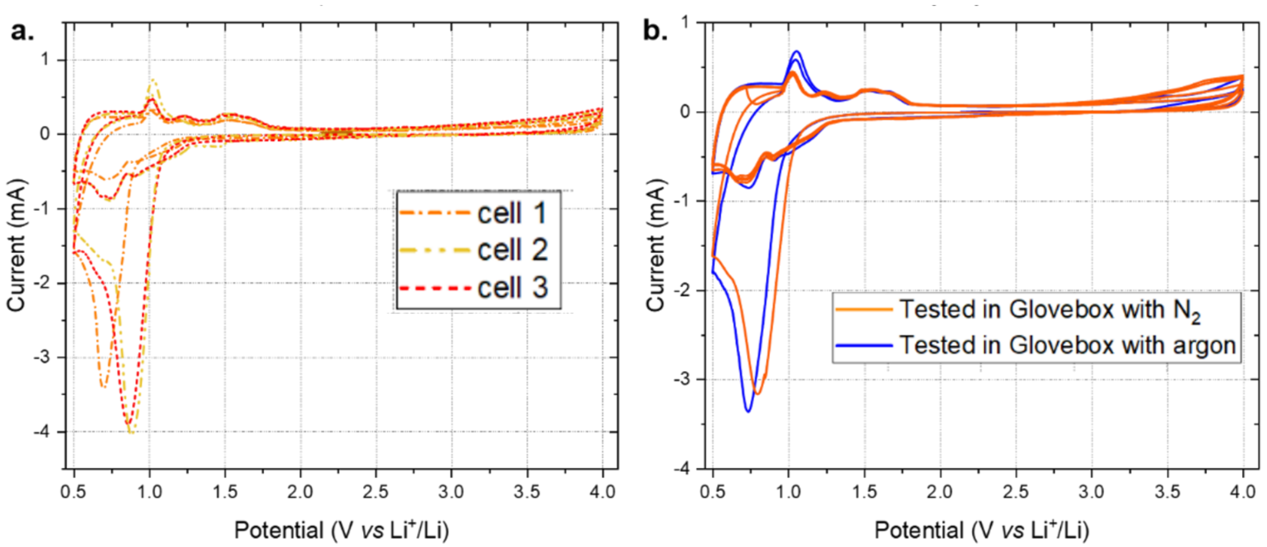

Figure S8: Repeatability of CVs at 0.1 mV s^{-1}. **a.** CV traces (1st and 2nd cycle) of three identical coin cells (in different colors) with a holed cap, tested in a N_2-filled glovebox, assembled with a lithium anode, 1.55 mm-thick GF separator soaked in $LiCF_3SO_3$ 1 M in TEGDME, and GDL as cathode. The cells were tested in an N_2-filled glovebox. **b**. CV traces (1st to 4th cycle) of cells with the same compositions, tested under argon *vs* N_2 environment.

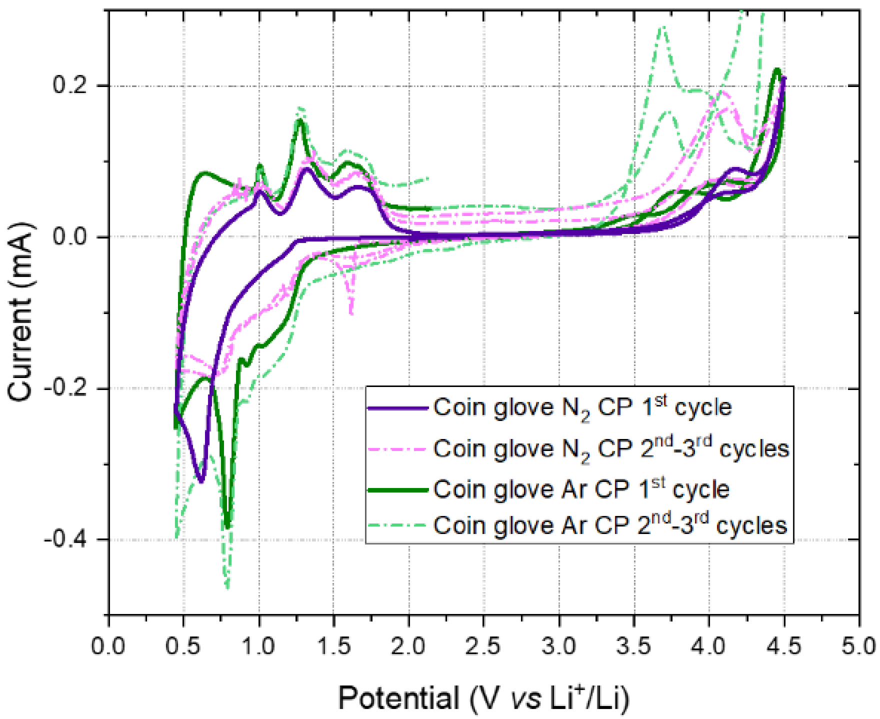

Figure S9: Repeatability of CVs with CP and at different scan rates for coin cells with a holed cap, tested in a glovebox, assembled with a lithium anode, 1.55 mm-thick GF separator soaked in $LiCF_3SO_3$ 1 M in TEGDME, and CP as cathode. CV (1st to 3rd cycle) at 0.05 mV s^{-1} for cells with CP as cathode, tested in argon (green) or N_2 (pink) environments.

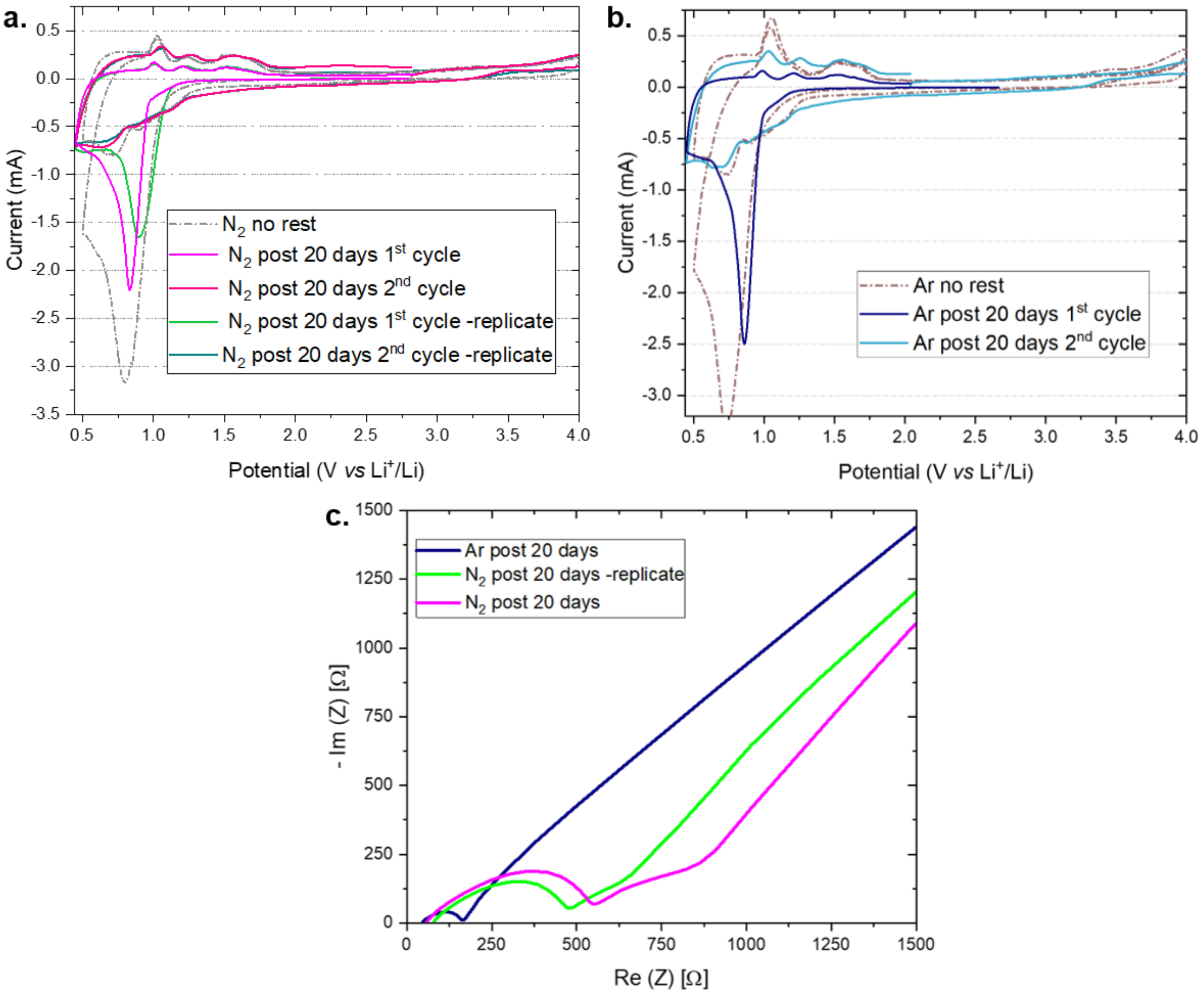

Figure S10: Repeatability of CV and EIS measurement on cells after 20 days of rest, performed in N_2 or argon. **a.** and **b.** CV traces at 0.1 mV s^{-1} of sealed coin cells assembled with a lithium anode, 1.55 mm-thick GF separator soaked in $LiCF_3SO_3$ 1 M in TEGDME, and GDL. **a.** CV traces obtained by testing, after the rest period, two different cells assembled in N_2 (green and pink lines), compared with a similar test without any rest period (grey line). **b.** CV traces of cells assembled in argon, performed after the rest period (blue line) or tested as prepared (grey line). **c.** EIS measurement of the two cells assembled in N_2 and the one assembled in argon, after 20 days of rest.

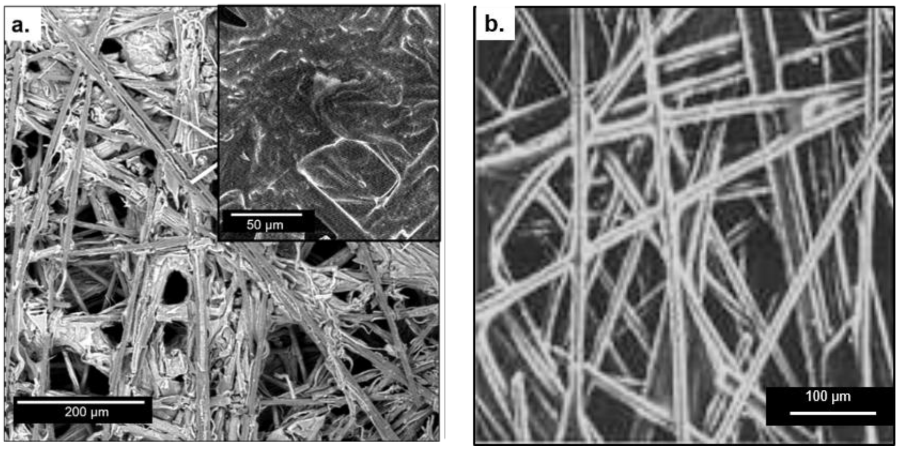

Figure S11: SEM analysis of post-mortem cathodes. **a**. CP cathode recovered after 3 cycles of CV, washed with a few drops of TEGDME, the same solvent used in the CV test. The white lines observed on the material surface were associated with residues of the GF separator. In the inset, a higher magnification of the same cathode obtained in a different area of the sample. **b**. Pristine CP from Toray Carbon Paper brochure.[1]

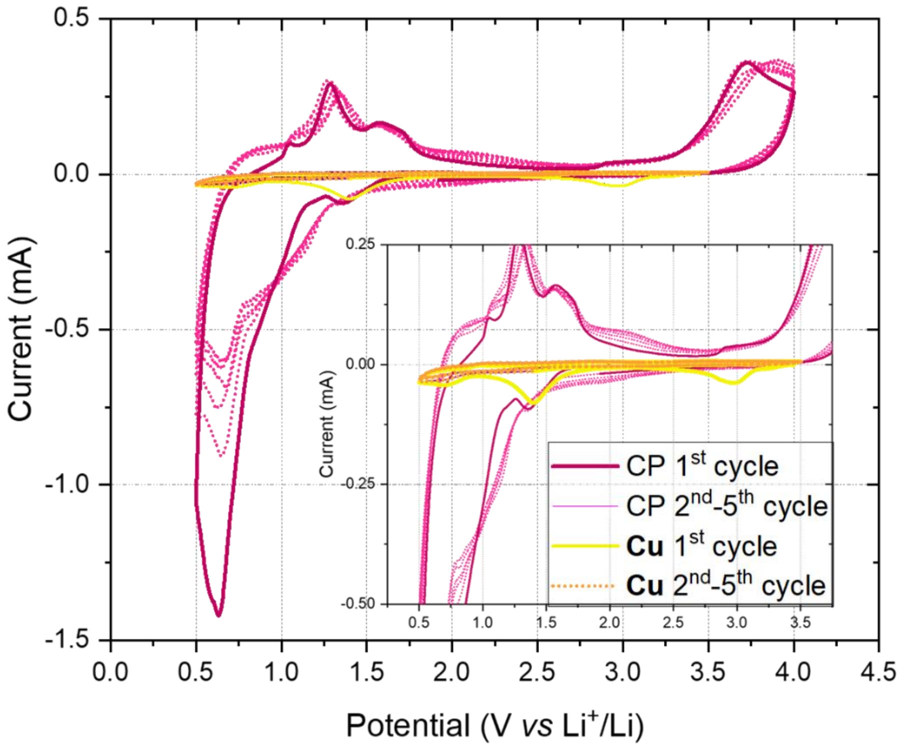

Figure S12: CV traces at 0.1 mV s^{-1} of EL-Cells assembled with a lithium anode, 1.55 mm-thick GF separator soaked in $LiCF_3SO_3$ 1 M in TEGDME, and CP (purple) or copper (orange) as cathode. The cells were tested by flowing 4 mL min^{-1} of filtered N_2 in the cell, and collecting the outlet in an acid trap with 8 mL of HCl 0.2 M. The inset shows the zoom of the CV with copper as a cathode.

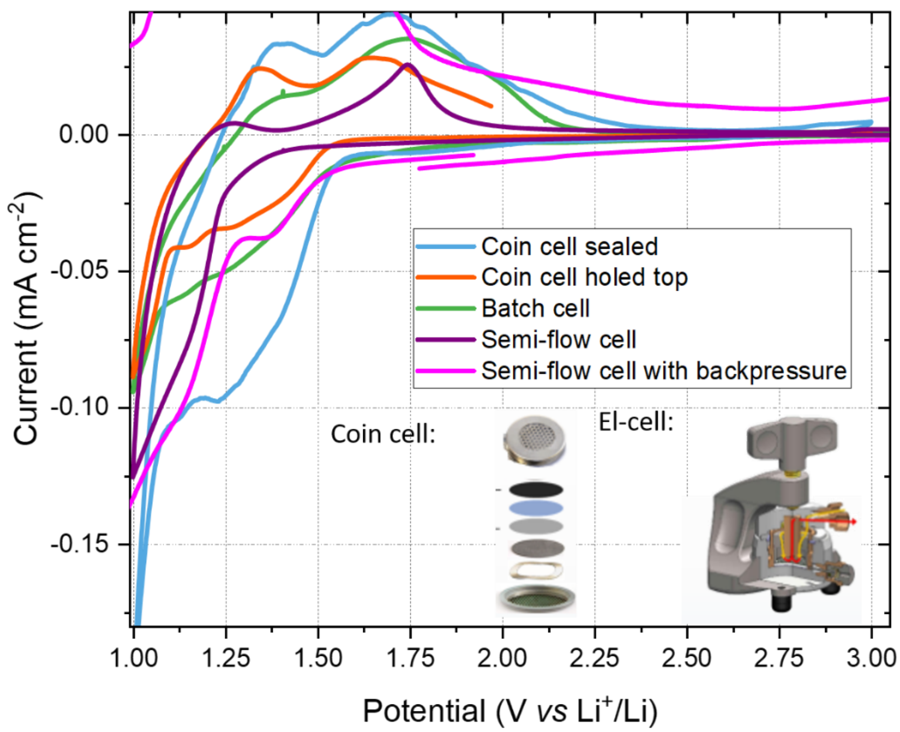

Figure S13: Second cycle of CV traces at 0.1 mV s^{-1} for different cell architectures, as explained in the legend. The cells were assembled with a lithium anode, 1.55 mm-thick GF separator soaked in $LiCF_3SO_3$ 1 M in TEGDME, and a carbon paper cathode. In the insets, the picture of the coin cell spare parts, on the left, and of an EL-Cell, on the right, with the gas flows indicated by the arrows.

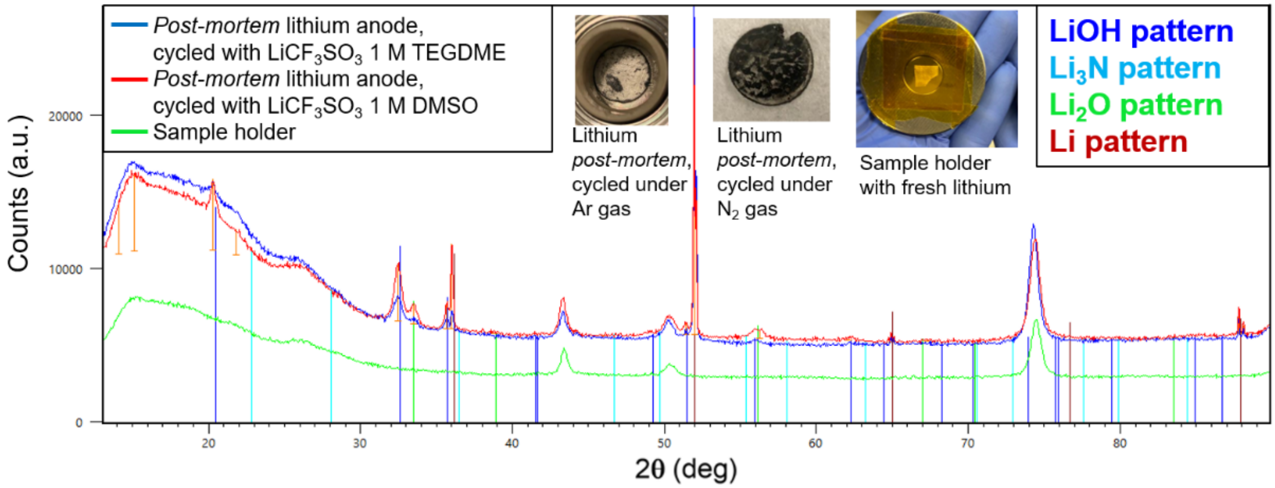

Figure S14: XRD spectra of lithium foils, used as anode, with two different electrolytes, after the CVs under N_2. The lithium anode electrodes, recovered from EL-Cells after the tests, were closed in the Kapton sample holder (photos on the right with a fresh sample of lithium inside). The patterns of the anodes tested with $LiCF_3SO_3$ 1 M in TEGDME (blue line) or DMSO (red line) were plotted together with the pattern of the sample holder itself (green). On the right, the legend reports the color of the respective species used to interpret the results. The patterns, following the color code, are shown in the plot for the chosen species and overlaid on the registered pattern. In the photos, the darkening of the lithium foil is notable in comparison with a lithium foil tested in the same setup, but under argon (photos on the left).

Table S1: Produced NH_3 (in μg cm^{-2} of geometric electrode area), calculated from the quantification of samples of acid traps and electrolytes obtained after 5 cycles of CV at 0.1 mV s^{-1} between 0.5 and 4 V *vs* Li^+/Li, operated in EL-Cell with a flow rate of 4 mL min^{-1} of different gas as specified in the table raw. The components of the cell and gas used are specified for each test.

**Cell name**	**Quantified NH_3 (μg cm^{-2})**		**Anode**	**Cathode**
	**Acid trap**	**Electrolyte**		
CV Li Ar	0.5	1	Li	CP
CV Li N_2	25	7	Li	CP

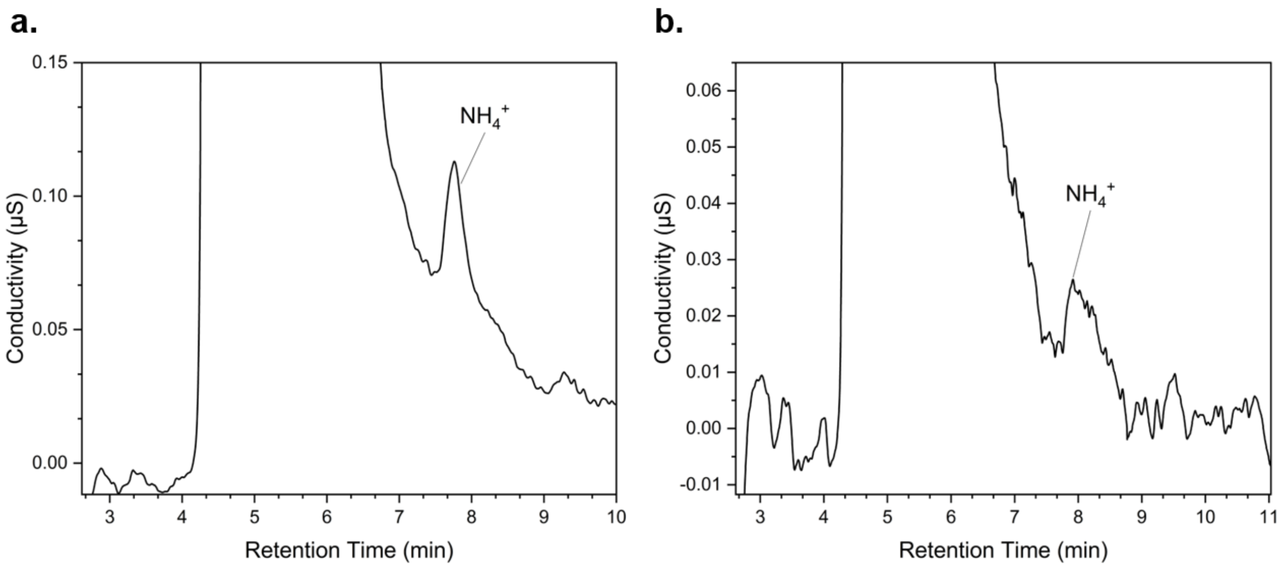

Figure S15: Chromatograms of two electrolyte samples collected after 5 cycles of CV at 0.1 mV s^{-1} between 0.5 and 4 V *vs* Li^+/Li, operated in EL-Cell semi-flow configuration, assembled with lithium as anode, CP as cathode, and $LiCF_3SO_3$ 1 M in TEGDME as electrolyte soaked in the GF separator. The flowed gas, send with a flow rate of 4 mL min^{-1}, was N_2 (**a**.) or argon (**b.**)

Bibliography

(1) *Toray Carbon Fiber Paper*, Fuel Cell Store. https://www.fuelcellstore.com/toray-carbon-paper-060
